# Safety and tolerability of tegoprubart in patients with amyotrophic lateral sclerosis: A Phase 2A clinical trial

**DOI:** 10.1371/journal.pmed.1004469

**Published:** 2024-10-31

**Authors:** Steven Perrin, Shafeeq Ladha, Nicholas Maragakis, Michael H. Rivner, Jonathan Katz, Angela Genge, Nicholas Olney, Dale Lange, Daragh Heitzman, Cynthia Bodkin, Omar Jawdat, Namita A. Goyal, Jeffrey D. Bornstein, Carmen Mak, Stanley H. Appel, Sabrina Paganoni

**Affiliations:** 1 Eledon Pharmaceuticals, Irvine, California, United States of America; 2 Departments of Neurology and Translational Neuroscience, St. Joseph’s Hospital and Medical Center and Barrow Neurological Institute, Phoenix, Arizona, United States of America; 3 Department of Neurology, The Johns Hopkins University School of Medicine, Baltimore, Maryland, United States of America; 4 Department of Neurology, Augusta University, Augusta, Georgia, United States of America; 5 California Pacific Medical Center Research Institute and Forbes Norris MDA/ALS Research and Treatment Center, San Francisco, California, United States of America; 6 Department of Neurology and Neurosurgery, Montreal Neurological Institute and Hospital, McGill University, Montreal, Canada; 7 Providence Portland Medical Center, Providence Brain and Spine Institute, Portland, Oregon, United States of America; 8 Department of Neurology, Hospital for Special Surgery, Weill Cornell School of Medicine, New York, New York, United States of America; 9 ALS Clinic, Texas Neurology, Dallas, Texas, United States of America; 10 Department of Neurology, University of Indiana, Indianapolis, Indiana, United States of America; 11 Department of Neurology, University of Kansas Medical Center, Kansas City, Kansas, United States of America; 12 Department of Neurology, University of California Irvine School of Medicine, Irvine, California, United States of America; 13 Mediar Therapeutics. Cambridge, Massachusetts, United States of America; 14 Department of Neurology, Houston Methodist Neurological Institute, Houston, Texas, United States of America; 15 Harvard Medical School, Sean M. Healey & AMG Center for ALS, Massachusetts General Hospital, Boston, Massachusetts, United States of America; Columbia University, UNITED STATES

## Abstract

**Background:**

The interaction of CD40L and its receptor CD40 on activated T cells and B cells respectively control pro-inflammatory activation in the pathophysiology of autoimmunity and transplant rejection. Previous studies have implicated signaling pathways involving CD40L (interchangeably referred to as CD154), as well as adaptive and innate immune cell activation, in the induction of neuroinflammation in neurodegenerative diseases. This study aimed to assess the safety, tolerability, and impact on pro-inflammatory biomarker profiles of an anti CD40L antibody, tegoprubart, in individuals with amyotrophic lateral sclerosis (ALS).

**Methods and findings:**

In this multicenter dose-escalating open-label Phase 2A study, 54 participants with a diagnosis of ALS received 6 infusions of tegoprubart administered intravenously every 2 weeks. The study was comprised of 4 dose cohorts: 1 mg/kg, 2 mg/kg, 4 mg/kg, and 8 mg/kg. The primary endpoint of the study was safety and tolerability. Exploratory endpoints assessed the pharmacokinetics of tegoprubart as well as anti-drug antibody (ADA) responses, changes in disease progression utilizing the Revised ALS Functional Rating Scale (ALSFRS-R), CD154 target engagement, changes in pro-inflammatory biomarkers, and neurofilament light chain (NFL).

Seventy subjects were screened, and 54 subjects were enrolled in the study. Forty-nine of 54 subjects completed the study (90.7%) receiving all 6 infusions of tegoprubart and completing their final follow-up visit. The most common treatment emergent adverse events (TEAEs) overall (>10%) were fatigue (25.9%), falls (22.2%), headaches (20.4%), and muscle spasms (11.1%). Mean tegoprubart plasma concentrations increased proportionally with increasing dose with a half-life of approximately 24 days. ADA titers were low and circulating levels of tegoprubart were as predicted for all cohorts. Tegoprubart demonstrated dose dependent target engagement associated and a reduction in 18 pro-inflammatory biomarkers in circulation.

**Conclusions:**

Tegoprubart appeared to be safe and well tolerated in adults with ALS demonstrating dose-dependent reduction in pro-inflammatory chemokines and cytokines associated with ALS. These results warrant further clinical studies with sufficient power and duration to assess clinical outcomes as a potential treatment for adults with ALS.

**Trial registration:**

Clintrials.gov ID:NCT04322149.

## Abstract 2

Steven Perrin and colleagues report on the safety and tolerability of different doses of the novel drug tegoprubart in patients with Amyotrophic Lateral Sclerosis.

## Author summary

**Why was this study done?:**

- One of the critical pathways controlling activation of T cells and B cells, cells which are pivotal in inducing inflammation in the body, involves a protein called CD40L and its receptor, CD40.
- Uncontrolled activation of CD40L/CD40 results in autoimmune disorders and the biological processes associated with organ transplant rejection.
- The activation of CD40L/CD40 has also been shown to occur in individuals with amyotrophic lateral sclerosis (ALS) as well as in animal models of ALS.
- Indeed, modulation of CD40L signaling ameliorates disease progression, improves survival, and decreases peripheral and neuroinflammation in rodent models of ALS.
- This study aimed to assess the safety and impact of an anti CD40L antibody, tegoprubart, on pro-inflammatory biomarkers in patients with ALS.

**What did the researchers find?:**

- We designed an open label, dose escalating study of tegoprubart in adults with ALS to assess the safety of tegoprubart at multiple doses and to study the effects of increasing doses of tegoprubart on inflammatory biomarkers in the blood.
- In this dose escalating study, tegoprubart appeared safe and well tolerated in 54 participants with ALS at all 4 doses studied.
- The amount of tegoprubart in blood increased with increasing dose.
- The study demonstrated target engagement with a dose-dependent decrease in the levels of CD40L and CXCL13.
- The study demonstrated a dose-dependent decrease in pro-inflammatory biomarkers after a single infusion of tegoprubart.

**What do the findings mean?:**

- This is the first clinical study of an anti CD40L antibody in ALS patients.
- The results suggest the inhibition of CD40L is safe and well tolerated and results in a dose-dependent reduction in a broad spectrum of T cell and myeloid associated pro-inflammatory signaling pathways in adults with ALS.
- These data support the further exploration of tegoprubart in a randomized, standard of care-controlled trial as a potential treatment for patients with ALS.
- There are 2 major limitations with the study:
  1. it is an open label study with no standard of care arm and the study was not powered to detect changes in clinical outcomes;
  2. the study was 12 weeks in duration which is not of sufficient duration to assess changes in clinical outcomes.

## Introduction

Amyotrophic lateral sclerosis (ALS) is a progressive neurodegenerative disease with a heterogeneous rate of disease progression and survival [1,2]. Disease heterogeneity is multifactorial and influenced by site of disease onset, age, genetics, rate of progression to time of diagnosis, respiratory function, and cognitive dysfunction [3]. There are 2 FDA approved treatments for ALS, Riluzole and Edaravone which may modulate disease progression in some populations of people with ALS [4–8].

Recent evidence suggests that some aspects of disease onset and progression in ALS are non-cell autonomous and regulated by microglia, astrocytes, oligodendrocytes, and peripheral immune cells. Microglial activation, astrocytosis, and the presence of infiltrating inflammatory cells have been well described in ALS. There is accumulation of IgG immunoreactive deposits in the spinal cord of ALS patients [9–13] and infiltration of lymphocytes [14,15], dendritic cells [16,17], monocytes, and macrophages into the spinal cord in ALS [18,19]. In addition, there is activation of the innate immune system in the periphery characterized by macrophage infiltration in skeletal muscle and phagocytosis of myelin sheaths on denervated nerves in animal models of ALS [20,21]. The activation of the innate immune system in animal models of ALS as well as in patients with ALS is mediated by the CD154/CD40 costimulatory pathway and specifically CD154 signaling [21]. Indeed, inhibition of CD154 signaling ameliorates disease progression, improves survival, and decreases peripheral and neuroinflammation in the SOD1 rodent model of ALS [21].

CD154 is a costimulatory type II membrane receptor found on activated T helper cells, platelets, endothelial cells, basophils, eosinophils, vascular smooth muscle cells, NK cells, astrocytes, and in some cases on B cells [22–28]. The receptor for CD154, CD40 is a transmembrane protein of the tumor necrosis factor receptor (TNFR) family found on antigen presenting cells (APCs) such as B cells, macrophages, dendritic cells, neutrophils, mesangial cells and tubular cells in the kidney, and microglia in the central nervous system [29–31]. The binding of CD154 to CD40 activates multiple downstream immune and inflammatory responses via the inhibition of effector and follicular T cell function, increased T regulatory function, inhibition of germinal center formation, inhibition of B cell maturation and antibody production, and inhibition of class switching [32–44]. The inhibition of CD40L has never been assessed in a clinical study in patients with ALS.

Tegoprubart is an immunoglobulin G1 (IgG1), kappa monoclonal antibody that blocks CD40 ligand (CD154) a costimulatory type II membrane receptor for CD40. CD40 is a costimulatory type I membrane protein found on APCs that is required for their activation. Tegoprubart has high affinity binding for CD154 and lacks Fc effector function thus mitigating risks of platelet activation and thromboembolisms. Tegoprubart is in clinical development for ALS and the prevention of transplant rejection.

Here, we report the results of an open label Phase 2A safety and tolerability study of tegoprubart in adults with ALS. Importantly, we demonstrate robust dose-dependent target engagement and reduction in pro-inflammatory biomarkers.

## Methods

### Trial design and oversight

This was an open-label, dose escalating trial conducted at 13 centers in the United States and Canada from November 2020 to May 2022 (clintrials.gov NCT04322149, https://clinicaltrials.gov/study/NCT04322149?cond=amyotrophic&intr=at-1501&rank=1). The trial was conducted in accordance with the Good Clinical Practice guidelines of the International Conference on Harmonisation and the ethical principles of the declaration of Helsinki. The study had a prospective protocol reviewed and approved by local Institutional Review Board or Ethics Committee at all trial sites. All participants provided written informed consent before any study related activities were initiated. The protocol is available (S1 PROTOCOL).

The trial was designed by the Sponsor, Eledon Pharmaceuticals in collaboration with the participating study site principal investigators. The Sponsor supplied Tegoprubart and was also involved in data analysis and in the preparation of the manuscript. An independent data monitoring committee reviewed the study data when 33% of each cohort had completed (i.e., after 3/9 participants had completed in the 1 mg/kg and 2 mg/kg cohorts and after 6/18 participants had completed in the 4 and 8 mg/kg cohorts).

### Trial participants

To be included in the trial, participants had to be adults with definite, probable, lab-supported probable, or possible ALS by revised El Escorial criteria, an ALS functional rating scale—Revised (ALSFRS-R) score of at least 35 with at least a total score of 9 on the 3 domains related to breathing and had to be within 24 months of their initial symptom onset. Concomitant use of the agents approved for ALS at the time of this trial, i.e., edaravone and riluzole, was permitted.

### Trial interventions and procedures

Participants meeting the eligibility criteria were dosed with tegoprubart via IV infusion every 2 weeks for a total of 6 infusions. This was the first multiple ascending dose (MAD) study of tegoprubart in humans. In a single ascending dose (SAD) in healthy volunteers, the half-life of tegoprubart was 14 to 24 days depending on dose. Cohort 1 (*n* = 9), 2 (*n* = 9), 3 (*n* = 18), and 4 (*n* = 18) received 1, 2, 4, and 8 mg/kg of tegoprubart, respectively. The cohorts were enrolled sequentially, and dosing could not begin in a higher dose cohort until the preceding cohort was fully enrolled, 33% of the cohort had completed dosing, and the Data Monitoring Committee (DMC) had reviewed the safety data from the cohort. Participants were infused every 2 weeks (on Day 1, 15, 29, 43, 57, and 71 +/− 2 days). At clinic visits, they also had their concomitant medications reviewed, reported any adverse events, had a symptom-directed physical examination, completed the Colombia Suicide Severity Rating Scale, the ALSFRS-R, and had blood drawn for safety, biomarkers, tegoprubart levels, and immunogenicity. In the weeks between clinic visits, participants had a telephone visit where concomitant medications were reviewed, and adverse events could be reported. A final visit occurred 4 weeks after the sixth infusion.

### Pharmacokinetic (PK) assay

Tegoprubart in human plasma was measured using a sandwich ELISA method. Briefly recombinant human CD40L (R&D Systems) were coated onto 96-well plates at 4 degrees. Plates were blocked with PBS/1%BSA to prevent nonspecific binding. Two-fold serial dilutions of test samples and AT-1501 (tegoprubart) reference standard were added to the plates and incubated at room temperature. Plates were washed and bound AT-1501 was detected with a peroxidase labeled donkey anti-human IgG (Jackson ImmunoResearch, PA) probe with color development using 3,3`,5,5`tetramethylbenzidine (TMB) substrate. The plates are read on a Molecular Devices SpectraMax 340 plate reader at an absorbance of 450 nm. Data for the titration curves are analyzed utilizing a 4-parameter logistic fit. The concentration of tegoprubart in the samples is determined from the AT-1501 standard curve.

### Trial outcomes

The primary objective of the study was to determine the safety and tolerability of IV administration of multiple doses of tegoprubart in adults with ALS. Exploratory objectives included: clinical outcome measures, including the change from baseline in ALSFRS-R, PK profiles, and anti-drug antibody (ADA) responses to tegoprubart. Additional exploratory assessments include an assessment of the effect of tegoprubart on pro-inflammatory biomarkers, as well as biomarkers of neurodegeneration, including neurofilament light chain (NFL).

### Protein biomarker profiling

Plasma protein profiling was performed at Myriad RBM (Austin, Texas, United States of America) using the HumanMAP multiplex Luminex array containing 94 protein analytes many of which are associated with inflammation and Simoa assays for NFL, TNF-α, and CXCL13.

### Statistical analyses

Descriptive statistics were provided for continuous variables and count and percentages for categorical variables. Participants’ demographics and disease characteristics were tabulated by cohort to assess compatibility. Decline in ALSFRS-R over the treatment period was based on subtracting the baseline value from the week 11 score, which necessitated the exclusion of 4 participants missing week 11 data. A post hoc least squared regression was also performed to estimate the individual ALSFRS-R slope during treatment for all participants as a sensitivity analysis. The intention to treat (ITT) population (*N* = 54) included all dosed participants. Data was analyzed by dose cohorts.

To assess the effect of tegoprubart on biomarkers, percent change in bioanalytical value from baseline was summarized by dose cohort. Statistical significance for each biomarker change was assessed via a mixed-model repeated measure (MMRM) accounting for dose and visits. The overall family-wise type 1 error was capped at 5% based on Holm–Bonferroni procedure [45].

## Results

### Trial participants

**Fig 1:**
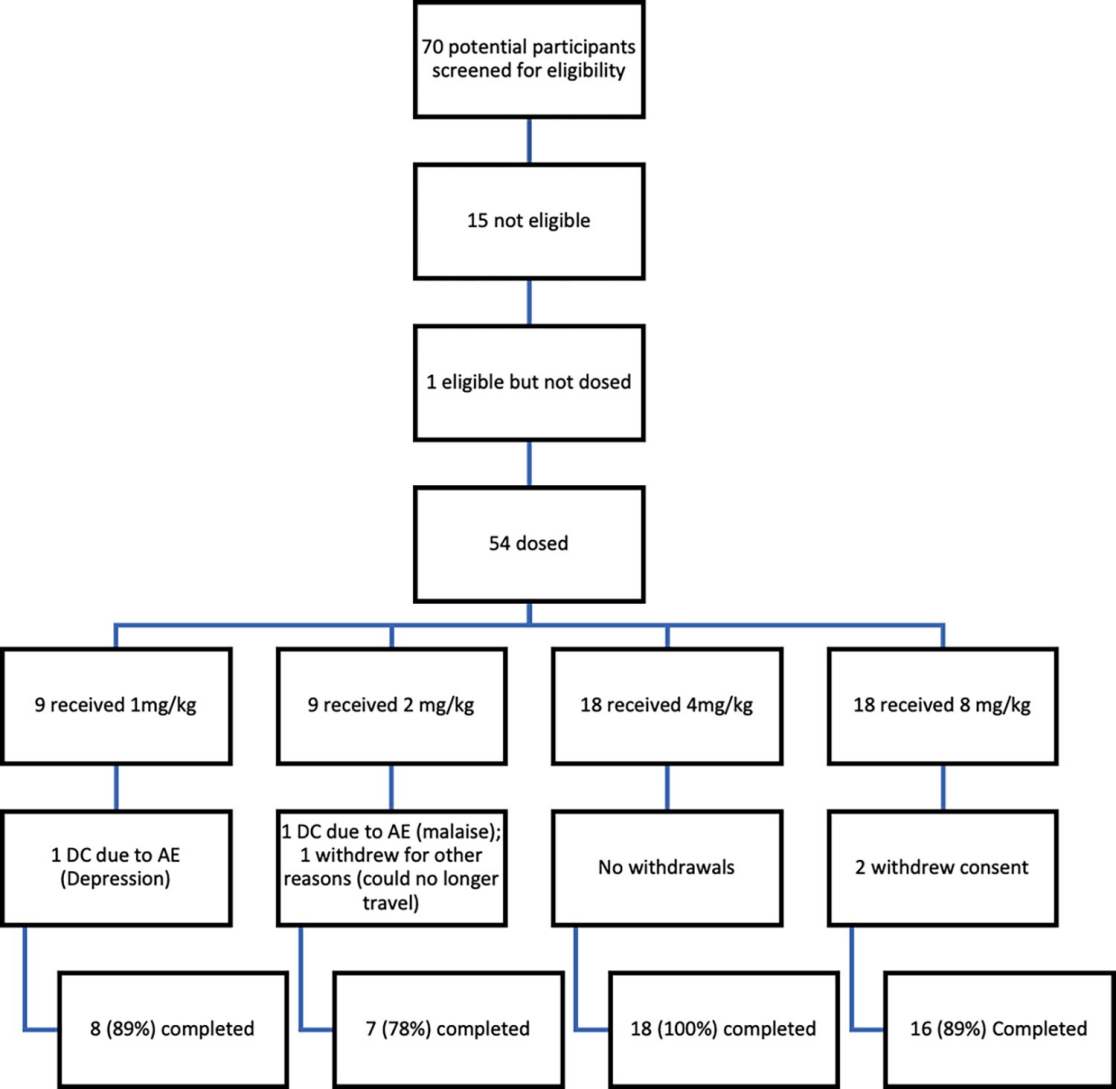
Participant disposition. Open label dose escalating study of AT-1501 in patients with ALS. Percentages reflect completed their last visit at week 12 and their post follow-up visit at week 15.

A total of 70 adults with ALS were screened for eligibility of whom 54 were included in the trial, 9 each in the 1 and 2 mg/kg cohorts and 18 each in the 4 and 8 mg/kg cohorts. As can be seen in Fig 1, 90.7% of all participants completed the trial. There were 2 protocol deviations in the study occurring at week 15 prior to end of study where 2 participants started prohibited concomitant medications (riluzole or dextromethorphan/quinidine). The demographics of the participants are summarized in Table 1. The median age of participants was 59.0 years and ranged from 39 to 85 years. There were nearly twice as many male participants (64.8%) as female participants (35.2%) and most participants were white (96.3%). There were more genetic mutations identified in the 8 mg/kg cohort (6 mutations (33%) including either SOD1, C9ORF72, or both) compared to the 1 mg/kg (1 mutation (11%) C9ORF72), no mutations in the 2 mg/kg cohort, and 1 C9ORF72 (5%) in the 4 mg/kg cohort.

**Table 1:** Cohort demographics and clinical characteristics.

Baseline characteristics	1 mg/kg (*N* = 9)	2 mg/kg (*N* = 9)	4 mg/kg (*N* = 18)	8 mg/kg (*N* = 18)	All (*N* = 54)
Age; mean (SD)	61.2 (6.50)	59.0 (14.8)	56.4 (8.53)	60.3 (11.1)	59.0 (10.3)
Age< 65; *N* (%)	7 (77.8)	6 (66.7)	15 (83.3)	11 (61.1)	39 (72.2)
Sex—male; *N* (%)	6 (66.7)	7 (77.8)	12 (66.7)	10 (55.6)	35 (64.8)
Hispanic/Latino; *N* (%)	0 (0)	0 (0)	0 (0)	3 (16.7)	3 (5.6)
White; *N* (%)	9 (100.0)	8 (88.9)	17 (94.4)	18 (100.0)	52 (96.3)
BMI; mean (SD)	26.2 (3.11)	27.6 (5.90)	25.1 (4.02)	26.0 (4.66)	26.0 (4.43)
Time (days) from: symptom onset mean (SD)	610.2 (340)	647.2 (389)	705.0 (580)	631.4 (431)	655.1 (457)
Time (days) from diagnosis mean (SD)	251.9 (242)	101.6 (75.3)	244.8 (204)	219.1 (175)	213.5 (189)
Total ALS-FRS; mean (SD)	40.4 (3.17)	40.0 (2.00)	39.6 (3.99)	38.6 (3.96)	39.5 (3.56)
Total ALS-FRS< = 34; *N* (%)	0 (0)	0 (0)	1 (5.6)	3 (6.7)	4 (7.4)
BULBAR DOMAIN< = 4; *N* (%)	0 (0)	0 (0)	2(11.1)	4 (22.2)	6 (11.1)
Rilutek	7 (77.8)	8 (88.9)	15 (83.3)	11 (61.1)	41 (75.9)
Edaravone	0 (0)	1 (11.1)	0 (0)	2 (11.1)	3 (5.6)

The baseline characteristics for participants in each dosing cohort: 1 mg/kg, 2 mg/kg, 4 mg/kg, 8 mg/kg, and all participants. SD, standard deviation; N, number; %, percentage.

ALSFRS-R Total Scores and Subdomain scores were comparable across treatment groups at Screening and Baseline. The median time since symptom onset and diagnosis were higher in the 1 and 2 mg/kg dose groups compared to the 4 and 8 mg/kg dose groups (Table 1). All study participants maintained their ALSFRS-R above the threshold score of 35 required for enrollment between the screening and baseline visits, with the exception of 1 participant in the 4 mg/kg group and 3 participants in the 8 mg/kg group who had aggregate ALSFRS-R scores of less than 35 at the time of first infusion. There was also a higher percentage of patients in the 4 (11%, *n* = 2) and 8 mg/kg (22%, *n* = 4) groups with a bulbar subdomain score less than 4 at time of first infusion, compared to the 1 and 2 mg/kg groups where there were no participants with a score of 4 or less. Three participants (1 in the 4 mg/kg group, 2 in the 8 mg/kg group) with a bulbar subdomain score of less than 4 also had aggregate ALSFRS-R scores less than 35 at the time of first infusion.

A total of 49 of the 54 participants (90.7%) completed the study. The 5 participants who discontinued the study received at least 4 doses of tegoprubart. Two participants discontinued due to treatment emergent adverse event (TEAEs) (1 participant with Grade 2 depression in the 1.0 mg/kg group; 1 participant with Grade 2 malaise in the 2.0 mg/kg group), 2 withdrew consent (both in the 8.0 mg/kg group), and 1 discontinued for the reason of Other (participant was undergoing treatment for tonsil cancer and missed the final visit).

### Safety assessment

A majority of participants had at least 1 TEAE (77.8% overall). The most common TEAEs overall (>10%) were fatigue (25.9%), falls (22.2%), headaches (20.4%), and muscle spasms (11.1%) (Table 2). Other common TEAEs overall (>5% to <10%) were arthralgia, dyspnea, dizziness, muscular weakness, contusion, depression, nausea, hypoesthesia, musculoskeletal stiffness, nasopharyngitis, and neck pain.

**Table 2:** Summary of TEAEs.

System organ class (SOC) preferred term (PT)	1.0 mg/kg (*N* = 9) *n* (%)	2.0 mg/kg (*N* = 9) *n* (%)	4.0 mg/kg (*N* = 18) *n* (%)	8.0 mg/kg (*N* = 18) *n* (%)	All (*N* = 54) *n* (%) *
Number of subjects with at least one TEAE	8 (88.9)	8 (88.9)	10 (55.6)	16 (88.9)	42 (77.8)
Nervous system disorders	3 (33.3)	5 (55.6)	5 (27.8)	7 (38.9)	20 (37.0)
Headache	3 (33.3)	2 (22.2)	3 (16.7)	3 (16.7)	11 (20.4)
Dizziness	2 (22.2)	1 (11.1)	1 (5.6)	1 (5.6)	5 (9.3)
Hypoaesthesia	1 (11.1)	2 (22.2)	0	0	3 (5.6)
General disorders and administration site conditions	4 (44.4)	5 (55.6)	0	9 (50.0)	18 (33.3)
Fatigue	4 (44.4)	3 (33.3)	0	7 (38.9)	14 (25.9)
Injury, poisoning, and procedural complications	1 (11.1)	3 (33.3)	6 (33.3)	6 (33.3)	16 (29.6)
Fall	0	3 (33.3)	5 (27.8)	4 (22.2)	12 (22.2)
Contusion	0	1 (11.1)	2 (11.1)	1 (5.6)	4 (7.4)
Musculoskeletal and connective tissue disorders	2 (22.2)	4 (44.4)	3 (16.7)	6 (33.3)	15 (27.8)
Muscle spasms	2 (22.2)	2 (22.2)	0	2 (11.1)	6 (11.1)
Arthralgia	0	1 (11.1)	2 (11.1)	2 (11.1)	5 (9.3)
Muscular weakness	1 (11.1)	1 (11.1)	1 (5.6)	2 (11.1)	5 (9.3)
Musculoskeletal stiffness	2 (22.2)	1 (11.1)	0	0	3 (5.6)
Neck pain	1 (11.1)	2 (22.2)	0	0	3 (5.6)
Gastrointestinal disorders	2 (22.2)	3 (33.3)	4 (22.2)	3 (16.7)	12 (22.2)
Nausea	0	1 (11.1)	1 (5.6)	2 (11.1)	4 (7.4)
Psychiatric disorders	4 (44.4)	3 (33.3)	0	3 (16.7)	10 (18.5)
Depression	2 (22.2)	1 (11.1)	0	1 (5.6)	4 (7.4)
Respiratory, thoracic, and mediastinal disorders	1 (11.1)	3 (33.3)	1 (5.6)	5 (27.8)	10 (18.5)
Dyspnoea	1 (11.1)	1 (11.1)	1 (5.6)	2 (11.1)	5 (9.3)
Infections and infestations	0	4 (44.4)	1 (5.6)	4 (22.2)	9 (16.7)
Nasopharyngitis	0	0	0	3 (16.7)	3 (5.6)

A summary table listing TEAEs.

* Includes TEAEs occurring in > 5% of total participants. N: number. Percentages are shown in parenthesis.

Common TEAEs (>5%) that were treatment related were headache (9.3%) and fatigue (9.3%), neither was dose proportional. All other treatment-related TEAEs were reported by 1 participant each and included dizziness, hypoesthesia, neuropathy peripheral, paresthesia, somnolence, asthenia, malaise, abdominal discomfort, abdominal pain upper, constipation, nasal congestion, productive cough, contact dermatitis, pruritus, flushing, hypertension, tinnitus, blood pressure increased, hyponatremia, depression, and suicidal ideation.

There were no deaths in this study; 1 participant had a serious adverse event (SAE) unrelated to treatment (tonsil cancer).

### Pharmacokinetics (PK) and anti-drug antibodies (ADA)

**Fig 2:**
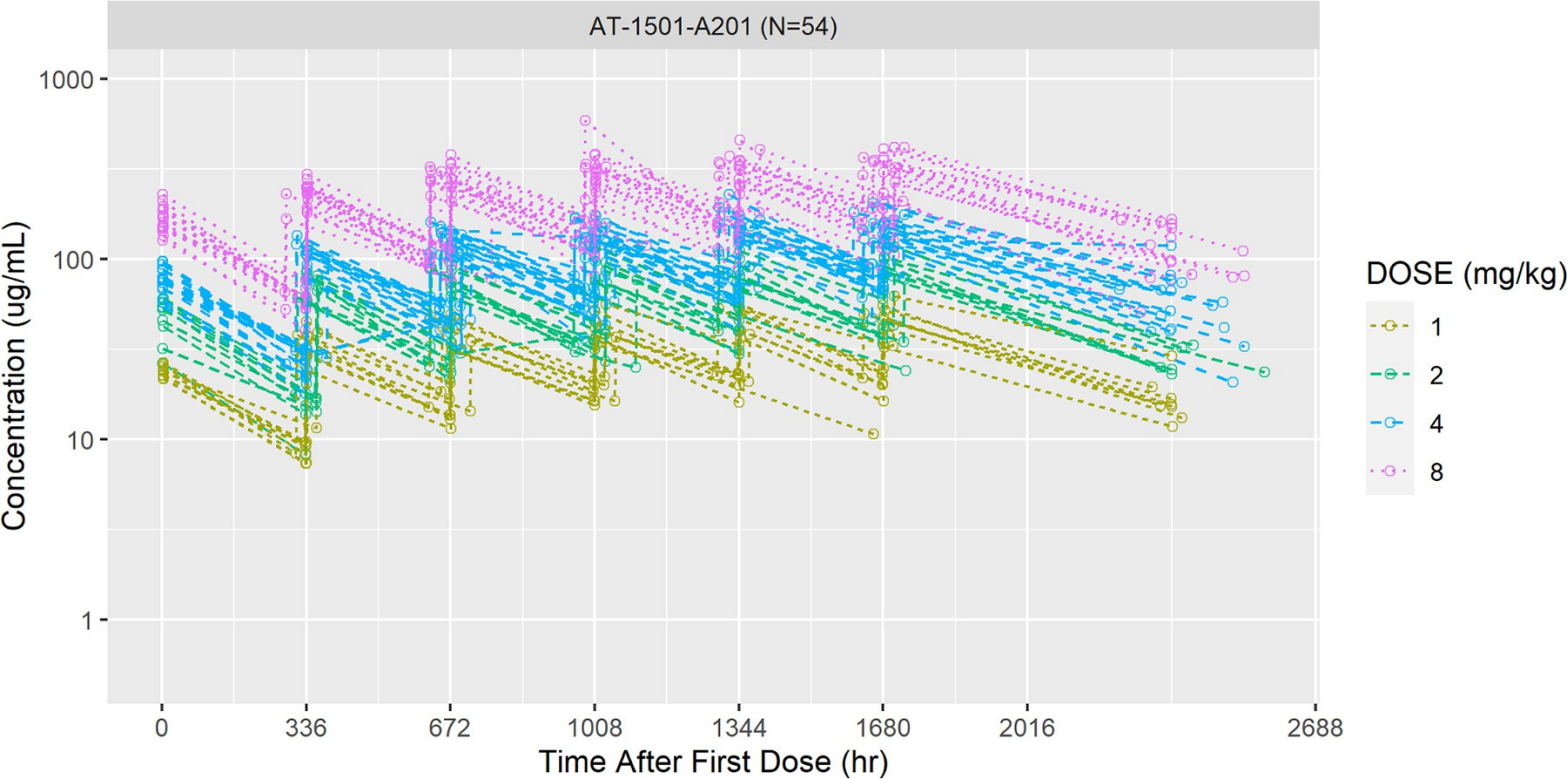
Plasma concentrations of AT-1501 over time in study AT-1501-A201 (Pharmacokinetic Population). Circulating concentrations of AT-1501 for each participant in each cohort: 1 mg/kg cohort (*n* = 9) gold; 2 mg/kg cohort (*n* = 9) green; 4 mg/kg cohort (*n* = 18) blue; 8 mg/kg cohort (*n* = 18) pink were determined by ELISA at the indicated time points. AT-1501 is the generic name for tegoprubart. AT-1501-A201 is the clinical study name for this Phase 2 study of tegoprubart in patients with ALS.

Overall, the PK of tegoprubart in this study was as predicted. Mean tegoprubart plasma concentrations increased proportionally with increasing dose (Fig 2). The half-life was approximately 24 days (median 505 to 634 h) and was similar for all 4 dose cohorts. The mean C_max_ and mean area under the curve (AUC) all approximately doubled with each doubling of the dose (Table 3). The AUC estimates total exposure of tegoprubart over time. The trough plasma concentration (C_trough)_ behaved in a similar fashion. Dose proportionality was achieved over the dose range of 1.0 to 8.0 mg/kg for C_max_ and AUC. Steady state was not reached, likely due to the doses being administered every 14 days while the half-life is approximately 24 days.

**Table 3:** Summary of plasma pharmacokinetic parameters of AT-1501 in study AT-1501-A201 (Pharmacokinetic Population).

		Mean	SD	Geomean	Range [min-max]	5^th^	95^th^
**1 mg/kg (Q2W)**							
	AUC_ss_	11,906	2,701	11,662	[8,501–18,111]	8,814	15,876
	C_max,ss_	46.5	15.6	42.3	[10.7–62.5]	21.1	60.5
**2 mg/kg (Q2W)**							
	AUC_ss_	21,536	3,083	21,347	[17,260–27,643]	17,947	26,263
	C_max,ss_	88	27.6	81.5	[24–117]	45.3	114.6
**4 mg/kg (Q2W)**							
	AUC_ss_	42,345	8,606	41,479	[25,176–56,437]	29,919	55,535
	C_max,ss_	161.4	27.3	159.4	[126–228]	129.4	207.6
**8 mg/kg (Q2W)**							
	AUC_ss_	81,252	16,826	79,544	[51,942–108,34]	53,844	107,682
	C_max,ss_	335.4	89.0	320.4	[114–457]	190	425.8

AUC_ss_ = area under the plasma concentration-time curve at steady state; C_max,ss_ = maximum plasma concentration at steady state; geomean = geometric mean; max = maximum; min = minimum; Q2W = every 2 weeks; SD = standard deviation.

*N* = 9, 9, 18, 18 for 1, 2, 4, 8 mg/kg. AUC_ss_ = DOSE/individual CL estimate from PPK model; C_max,ss_ = observed maximum concentrations from last dose phase; AUC in μg*hr/ml, C_max_ in μg/ml.

Positive ADA responses to tegoprubart were observed in 9 of 215 samples, detectable in 7 study participants (less than 5% of total samples). Four of the 9 ADAs were detected in 4 participants at the Week 15/End of Study time point. One participant in the 2.0 mg/kg group had detectable ADA titers prior to their first infusion of tegoprubart on Day 1. No other participants had detectable titers at this baseline assessment. There was no dose dependence with respect to the incidence of positive ADA titers. The ADA antibody titers were low and did not impact circulating levels of tegoprubart.

### Biomarker results

The study included 2 biomarker objectives: (1) Target engagement assessing the levels of CD40L, CD40, and CXCL13 from Day 71 to the average levels at screening and baseline; (2) pro-inflammatory response assessing the circulating levels of 35 pro-inflammatory biomarkers at Day 71 to the average of screening and baseline.

**Fig 3:**
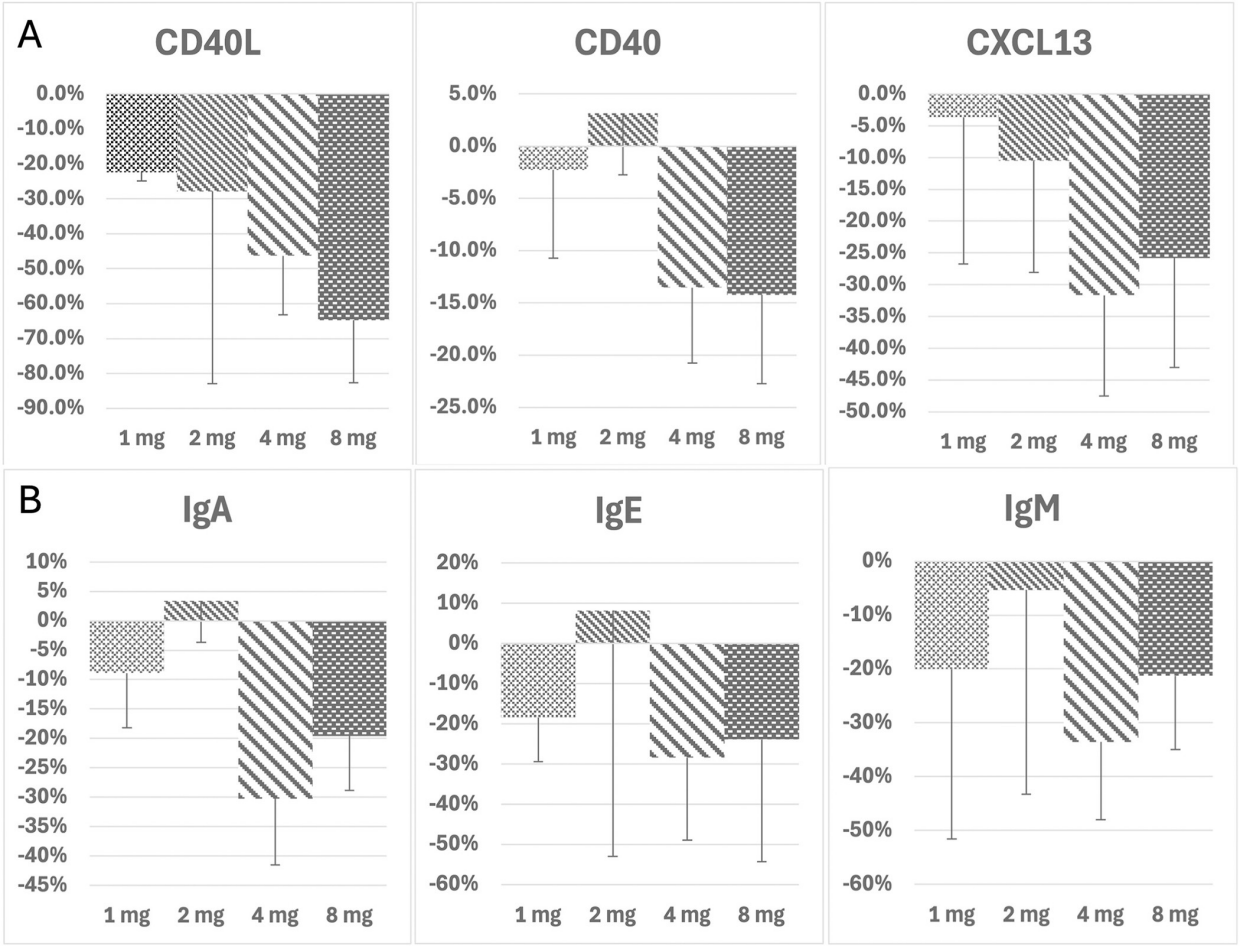
Biomarkers of target engagement. (A, B) The axis is dose in mg/kg. The Y axis is the percent mean reductions in protein biomarkers from baseline to Day 71. Error bars are standard error of the mean for participants in each cohort respectively.

There were dose-dependent reductions in CD154, CXCL13 and CD40 levels at Day 71 (the final on-treatment assessment) compared to the average of screening and baseline levels (Fig 3A). The largest reductions were seen at the 4 and 8 mg/kg doses. The highest reductions were observed for CD154, the target of tegoprubart on T cells, where levels were reduced by 46.14% and 64.7% at the 4 and 8 mg/kg doses at 71 days, respectively. Reductions in CD154 translated to reductions in CXCl13 (32.05% and 25.79%) and CD40 (13.53% and 14.22%) at the 4 and 8 mg/kg doses at day 71, respectively (Fig 3A and Table 4).

**Table 4:** Percent changes in the levels of pro-inflammatory biomarkers at 15 and 71 days from baseline.

Protein Mean %Change (*N*, SD)	Dose	Day 15	Day 71
AMYLOIDP(SAP)*	4 mg/kg	−8.38 (18, 13.98)	−10.80 (18, 17.05)
AMYLOIDP(SAP)*	8 mg/kg	−9.81 (18, 20.08)	−18.66 (16, 13.42)
B2M*	4 mg/kg	−10.07 (18, 11.78)	−16.99 (18, 10.73)
B2M*	8 mg/kg	−13.43 (18, 12.55)	−16.57 (16, 13.30)
C3*	4 mg/kg	−9.61 (18, 19.33)	−28.99 (18, 8.94)
C3*	8 mg/kg	−12.86 (18, 13.64)	−14.87 (16, 11.86)
CD40*	4 mg/kg	−6.51 (18, 10.36)	−13.53 (18, 14.43)
CD40*	8 mg/kg	−13.02 (18, 11.40)	−14.22 (16, 17.02)
CD40L (CD154)*	4 mg/kg	−74.79 (18, 14.25)	−46.14 (18, 34.42)
CD40L (CD154)*	8 mg/kg	−78.50 (18, 26.53)	−64.70 (16, 35.72)
CXCL10	4 mg/kg	−18.33 (18, 22.77)	−15.32 (18, 24.06)
CXCL10*	8 mg/kg	−18.01 (18, 15.72)	−24.03 (16, 21.26)
CXCL13*	4 mg/kg	−22.24 (18, 14.36)	−32.05 (18, 15.20)
CXCL13*	8 mg/kg	−26.74 (18, 14.63)	−25.79 (16, 17.25)
CXCL9*	4 mg/kg	−20.57 (18, 28.38)	−24.96 (18, 32.48)
CXCL9*	8 mg/kg	−25.26 (18, 22.82)	−37.74 (16, 20.40)
IgA*	4 mg/kg	−11.78 (18, 17.44)	−30.24 (18, 11.34)
IgA*	8 mg/kg	−11.81 (18, 11.97)	−19.58 (16, 9.40)
IgE*	4 mg/kg	−14.76 (18, 23.24)	−28.62 (18, 20.85)
IgE*	8 mg/kg	−12.22 (18, 15.88)	−23.95 (16, 29.38)
IgM*	4 mg/kg	−12.53 (18, 17.84)	−33.57 (18, 14.45)
IgM*	8 mg/kg	−12.77 (18, 17.35)	−21.29 (16, 13.69)
IL2RA*	4 mg/kg	−12.89 (18, 11.44)	−25.11 (18, 11.50)
IL2RA*	8 mg/kg	−21.13 (18, 14.48)	−30.39 (16, 12.93)
IL-16*	4 mg/kg	−5.63 (18, 12.95)	−19.21 (18, 9.04)
IL-16*	8 mg/kg	−13.10 (18, 12.02)	−12.73 (16, 25.00)
MDC (CCl22)*	4 mg/kg	−14.44 (18, 15.38)	−16.40 (18, 11.47)
MDC (CCl22)*	8 mg/kg	−12.10 (18, 22.70)	−8.60 (16, 30.23)
MMP2*	4 mg/kg	−6.16 (18, 10.66)	−13.41 (18, 7.98)
MMP2*	8 mg/kg	−9.48 (18, 16.91)	−14.67 (16, 20.50)
TNF-alpha*	4 mg/kg	−12.32 (18, 14.82)	−23.94 (18, 15.57)
TNF-alpha*	8 mg/kg	−9.91 (18, 20.63)	−20.40 (16, 21.05)
TNFSR2*	4 mg/kg	−12.27 (18, 14.53)	−19.25 (18, 15.62)
TNFSR2*	8 mg/kg	−22.48 (18, 20.17)	−30.74 (16, 10.57)
VCAM1*	4 mg/kg	−15.61 (18, 14.31)	−29.01 (18, 9.00)
VCAM1*	8 mg/kg	−13.00 (18, 9.63)	−16.18 (16, 10.05)

Reductions in pro-inflammatory biomarkers from baseline to Day 15 and Day 71, respectively. *N*: number of participants. SD: standard deviation.

* Statistically significant reduction from baseline at Day 71. Overall family-wise error is controlled at <5%.

Other proteins associated with costimulatory activity were also assessed including changes in IgM, IgA, and IgE levels as blocking CD154 prevents class switching [35,38]. There were dose-dependent reductions in IgA, IgM, and IgE, with maximal reduction in the 4 and 8 mg/kg cohorts (Fig 3B). In addition, dose escalation resulted in a higher percentage of participants in each dose escalating cohort demonstrating a decrease in each respective biomarker. Interestingly, decreases in expression of CD154, CD40, CXCL13, IgM, IgA, and IgE were observed at the Day 15 visit (i.e., after just a single infusion of tegoprubart) suggesting rapid engagement of target and downstream cellular responses such as antibody production (Table 4).

A multiplex Luminex assay was utilized to assess the expression of 91 proteins as well as Simoa assay to analyze the expression of TNF-α, IL-6, and NFL. 35 proteins with sufficient data (>80% data above LOQ) and with relevance to immune function based on Gene Ontology (GO) were considered for further analysis.

**Fig 4:**
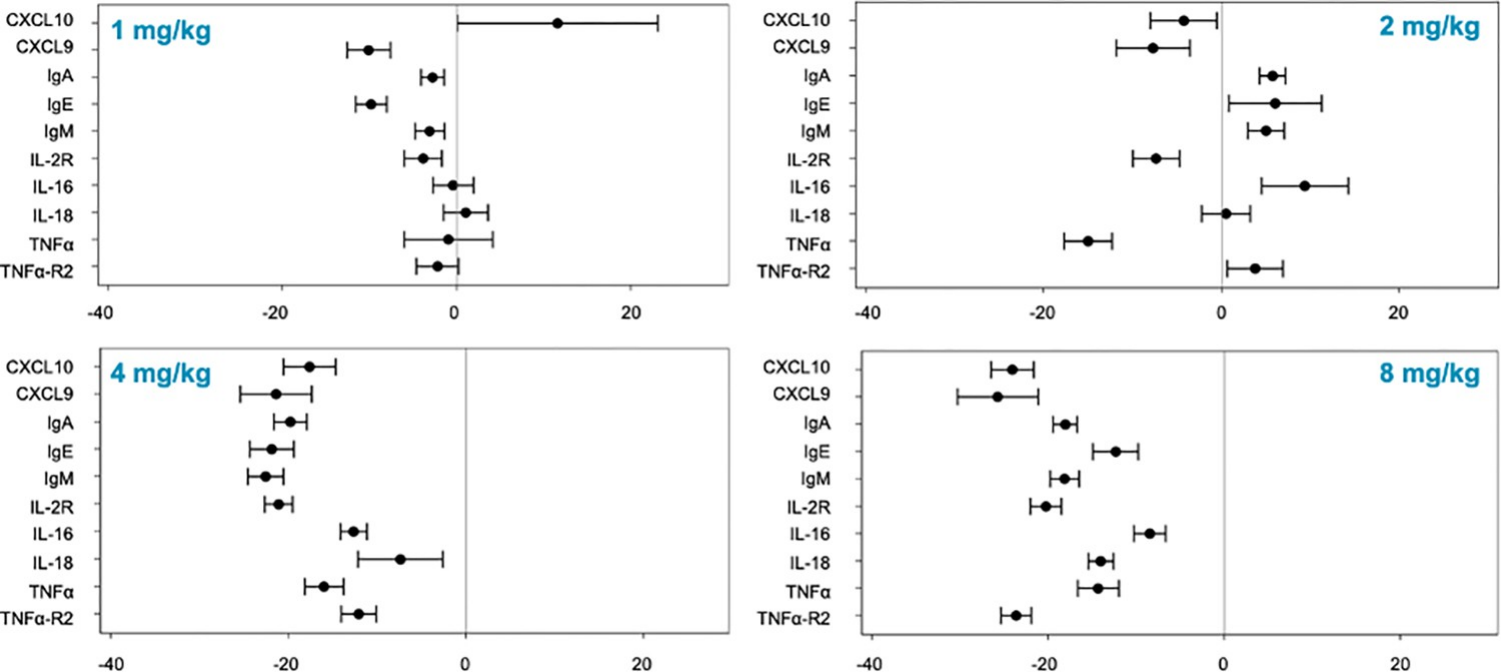
Mean changes (+/− SEM) in pro-inflammatory proteins by tegoprubart dose groups. Forest plots for dose-dependent reduction of pro-inflammatory biomarkers. Forest plots graph the percent mean reductions in protein biomarkers from baseline to Day 71 on the X axis. Error bars are standard error of the mean for participants in each cohort, respectively.

Tegoprubart reduced the levels of CD154 on T cells and CD40 on B cells and APCs suggesting effective inhibition of costimulatory signaling on T cells and APCs resulting in statistically significant decreases of multiple downstream pro-inflammatory proteins with diverse biological functions including B2M, C3, CCL22, CXCL9, CXCL10, IL2ra, IL-16, IL-18, TNFα, TNFR2, SAP, and VCAM1 with the highest reduction being observed in the 4 and 8 mg/kg doses at Day 71 compared to baseline levels (Fig 4 and Table 4). Percent reductions were of similar magnitude to what was observed for CD40 and CXCL13 ranging from −12.73% for Il-16 to −37.74% for CXCL9 (Table 4) in the 8mg/kg dose.

The apparent increase in CXCL10 at the 1 mg/kg dose with large standard deviations was driven by a single participant that had a greater than 20% increase in CXCL10 from 12 weeks to baseline. Similarly, the increases in IL-16, the Igs and TNFα-R at the 2 mg/kg dose could be due to the small sample size and would need to be further characterized in larger study.

### Neurofilament light chain

The levels of NFL were assessed at baseline and at Day 71. The mean percent change in NFL levels for the 1 mg/kg cohort was 3.8% (+/− 0.07), 2 mg/kg cohort 3.8% (+/− 0.19), 4 mg/kg cohort 0.9% (+/− 0.13), and for the 8 mg/kg cohort −0.7% (+/− 0.10). Although there were no apparent changes in NFL levels associated with tegoprubart treatment, higher levels of NFL at screening did correlate with faster disease progression as has been described previously in other studies [46].

### Change in ALS functional rating scale (ALSFRS-R)

Decline in ALSFRS-R was assessed using a least squares mean slope model (LSM). The monthly decline in ALSFRS-R was −0.99 for the entire population, which is a rate similar to the natural history in ALS (Table 5). It would appear that the progression rate was faster in the 8 mg/kg cohort, but this could be due to the small sample size or variability in patient demographics for this particular cohort.

**Table 5:** Least mean squares monthly change in ALSFRS-R.

AT-1501	Mean (STD, *N*)
All participants	−0.99 (0.20; 54)
1 mg/kg	−0.3 (0.34, 9)
2 mg/kg	−0.83 (0.38, 9)
4 mg/kg	−0.57 (0.32, 18)
8 mg/kg	−1.83 (0.39, 18)

The monthly change in ALSFRS-R for each dosing cohort. STD: standard error. N: number of participants.

## Discussion

This Phase 2A study of tegoprubart in adults with ALS was the first clinical study assessing the inhibition of CD40L in this patient population. We achieved the primary endpoint of safety and tolerability of tegoprubart up to 8.0 mg/kg which appeared to be safe and well tolerated in this ALS population. Most TEAEs were mild to moderate in severity, and most participants received the full treatment regimen as planned. The most common TEAEs overall (>10%) were fatigue, falls, and muscle spasms, which are expected events in this ALS population. This was the first MAD study of tegoprubart in humans. We selected a dosing frequency of every 2 weeks based on limited pharmacokinetic data from a SAD study in healthy volunteers. In this study, the pK of tegoprubart was linear and dose proportional with a half-life consistent with every 3-week dosing in future studies. ADAs were seen infrequently and at low titers. ADAs did not impact serum concentration of tegoprubart. This was the first clinical study assessing the inhibition of CD40L signaling in ALS patients.

Tegoprubart is an anti CD154 antibody designed to inhibit innate and adaptive immune responses, and animal model data supports that blocking CD154 with tegoprubart can alter both the inflammatory component of ALS and its progression.

In this clinical study, tegoprubart administration resulted in a dose-dependent reduction in the levels of CXCL13, CD154, and CD40 as well as an increase in the number of participants with decreased levels of these biomarkers. CD154 is the direct target of tegoprubart and expressed on the cell surface of activated T cells and shed from the cell surface as a functional cytokine, soluble CD40 ligand (sCD40L or sCD154). CD154 was reduced significantly at all dose levels and these reductions were rapid occurring by day 15 and sustained through day 71. CD40 is the receptor for CD154 constitutively expressed on the cell surface of B cells, macrophages, dendritic cells, NK cells, and specialized APCs [22–28]. The levels of CD40 were reduced by 6.51% and 13.02% by day 15 at the 4 and 8 mg/kg doses, respectively, and levels continued to be reduced by greater than 10% by day 43 and out to day 71. The magnitude of reduction in CD40 levels are difficult to interpret in regards to immune cell activation for 2 reasons. CD40 is ubiquitously expressed on many immune cell types that may not be directly involved in costimulatory signaling and B cell maturation. In addition, CD40 is constitutively expressed on the cell surface of these cell types thus specific changes in the levels of CD40 on B cells may be difficult to assess. In order to assess germinal center formation and B cell maturation more directly, we assessed the levels of CXCL13. CXCL13 is a chemokine expressed by follicular dendritic cells and follicular T cells (Tfh) in the light zone of the germinal center after antigen presentation to Tfh [39–40]. The expression of CXCL13 chemotactically recruits CXCR5 expressing B cells to the light zone and in conjunction with CD154 expression on activated T cells and CD40 expression on the recruited B cell results in the preliminary phase of B cell antigen presentation and maturation [40]. The levels of CXCL13 were reduced by more than 20% by day 15 at the 4 and 8 mg/kg dose levels and these levels of reduction were maintained out to 71 days. These data suggest robust target engagement by tegoprubart after the first IV infusion with significant reductions by day 15.

Target engagement resulted in a rapid reduction in pro-inflammatory markers by day 15 with 10% to greater than 20% reductions in pro-inflammatory proteins associated with T cell activation (Il2ra, CD40L, CCL22, IL16), B cell maturation (IgA, IgE, IgM, CD40), acute response (SAP, VCAM1), chemotaxis (CXCL13, CXCL10, TNFα), and antigen presentation (B2M, CXCL9), and these reductions were maintained through day 71 (Table 4). For many pro-inflammatory biomarkers including biomarkers of target engagement, there was some plateau in reduction of levels between the 4 and 8 mg/kg dose levels. The plateau suggests we have identified a reasonable dose range for subsequent clinical studies.

In the last 10 years, it has been recognized that the peripheral immune system plays a role in the activation of neuroinflammation in the CNS. Macrophage accumulation and phagocytosis of denervated nerves leads to neuromuscular junction loss and muscle atrophy in animal models of ALS. Indeed, deletion of pro-inflammatory peripheral macrophages or the deletion of T effector cells, or Treg supplementation improve survival and slows disease in animal models of ALS [21,47–50]. Pharmacologically the inhibition of CD154 signaling in the SOD1^G93A^ preclinical model reduces macrophage accumulation on peripheral nerves, increases neuromuscular junction occupancy, decreases microglial and astrocyte activation, and improves motor neuron survival [21].

This Phase 2 study was of short duration and not designed or powered to detect changes in the rate of ALSFRS-R decline over 71 days. There were no apparent dose related changes in ALSFRS-R in the study population. The faster rate of decline in the 8 mg/kg cohort could be attributed to the higher percentage of participants having genetic (SOD1, C9ORF72 expansion) mutations in this cohort (33%) compared to the 11%, 0%, and 5% in the 1, 2, and 4 mg/kg cohorts, respectively. There was also a higher percentage of patients in the 4 mg/kg (11%, *n* = 2) and 8 mg/kg (22%, *n* = 4) groups with a bulbar subdomain score less than or equal to 4 at time of first infusion, compared to the 1 and 2 mg/kg groups where there were no participants with a score of 4 or less. Three participants (1 in the 4 mg/kg group, 2 in the 8 mg/kg group) with a bulbar subdomain score of less than 4 also had aggregate ALSFRS-R scores less than 35 at the time of first infusion.

There were several limitations in the current study. The study was designed as an open label study with no placebo group and had broad inclusion/exclusion criteria since the primary endpoint of the study was safety and tolerability. Furthermore, the study was not a placebo-controlled study, was not designed or powered and not of sufficient duration to assess changes in disease progression measured by ALSFRS-R due to patient heterogeneity and the variability of changes in ALSFRS-R in short studies. The duration of study may have also impacted the inability to see changes in NFL associated with other biomarker changes. However, levels of NFL at time of enrollment did correlate with disease progression rate as has been described in other ALS studies. In addition, there was a higher than normal prevalence of white subjects enrolled in the study which may impact outcomes in future studies.

The strength of this study demonstrated tegoprubart was able to reduce the levels of pro-inflammatory biomarkers in a dose-dependent fashion. Several of these proteins can be considered as indirect measures of target engagement, and these were the ones most greatly reduced by tegoprubart.

## Conclusion

The data from this Phase 2 study shows that tegoprubart appeared to be safe and well tolerated at the doses tested in this patient population and treatment with tegoprubart demonstrated robust target engagement and a decrease in T cell and myeloid pro-inflammatory signaling. The data support further investigation of tegoprubart in well-powered placebo-controlled studies in patients with ALS.

## Supporting information

S1 CONSORT DEFINE CHECKLIST Consort-Define downloadable checklist. (DOCX)

S1 PROTOCOL Clinical trial protocol. (DOCX)

## Abbreviations

ADA: anti-drug antibody
ALS: amyotrophic lateral sclerosis
APC: antigen presenting cell
AUC: area under the curve
DMC: Data Monitoring Committee
GO: Gene Ontology
ITT: intention to treat
MAD: multiple ascending dose
MMRM: mixed-model repeated measure
NFL: neurofilament light chain
SAD: single ascending dose
SAE: serious adverse event
TEAE: treatment emergent adverse event
TNFR: tumor necrosis factor receptor
