# Supplementary material for: Safety and tolerability of tegoprubart in patients with amyotrophic lateral sclerosis: A Phase 2A clinical trial
Source: PLoS Med. 2024 Oct 31;21(10):e1004469. doi: 10.1371/journal.pmed.1004469 (PMC11527214; doi:10.1371/journal.pmed.1004469)
Supplement: S1 CONSORT DEFINE CHECKLIST — (DOCX) [file pmed.1004469.s001.docx]

# Web appendix 2: CONSORT-DEFINE downloadable checklist

**Recommended checklist items to consider in an early phase dose-finding clinical trial report from CONSORT 2010 and CONSORT-DEFINE checklists**^^^

Please cite as: Yap C, Solovyeva O, de Bono J, et al. Enhancing reporting quality and impact of early phase dose-finding clinical trials: CONSORT Dose-finding Extension (CONSORT-DEFINE) guidance. *BMJ* 2023;383:e076387. doi:10.1136/bmj-2023-076387

| **Category and section** | **Standard CONSORT 2010**  **checklist item** | | **CONSORT-DEFINE checklist item**  **for EPDF Trials** | | **Addressed on Page No^¤^** |
| --- | --- | --- | --- | --- | --- |
|  | **Item No** | **CONSORT 2010** | **Item**  **No** | **CONSORT-DEFINE** |  |
| **Title and abstract** | | | | | |
|  | 1a | Identification as a randomised trial in the title | 1a† | Identification as an early phase dose-finding (eg, first-in-human, dose escalation or de-escalation, phase 1, phase 1/2, expansion, dose titration) and, if applicable, randomised trial in the title or abstract | Title, paragraph 1 |
|  | 1b | Structured summary of trial design, methods, results, and conclusions (for specific guidance, see CONSORT for abstracts) | 1b | Structured summary of trial design, methods, results, and conclusions (for specific guidance, see CONSORT-DEFINE for abstracts) | Methods and Findings paragraphs 2, 3; Conclusions paragraph4 |
| **Introduction** | | | | | |
| Background  and objectives | 2a | Scientific background and explanation of rationale | 2a.1† | Description of research question(s) and justification for undertaking the trial, including summary of relevant clinical studies (published and unpublished) examining benefits and harms for each intervention | Author Summary paragraph 1; Introduction paragraph 3 and 4 |
|  |  |  | 2a.2* | Summary of key findings from relevant non-clinical or preclinical research | Introduction paragraph 2 |
|  |  |  | 2a.3* | Summary of findings from previously generated preclinical and translational studies to support any planned biomarker substudies (where applicable) | Introduction paragraph 3 |
|  | 2b | Specific objectives or hypotheses | 2b† | Specific objectives (eg, relating to safety, activity, pharmacokinetics, pharmacodynamics, recommended dose(s)) | Introduction paragraph 4 |
| **Methods** | | | | | |
| Trial design | 3a | Description of trial design (such as parallel, factorial) including allocation ratio | 3a.1† | Description of trial design elements, such as dose escalation or de-escalation strategy, number of treatment groups, allocation ratio if relevant, and details of any prespecified trial adaptations | Methods Trial Design and Oversight paragraph 1; Methods Trial Interventions and Procedures paragraph 4 |
|  |  |  | 3a.2* | Trial design schema to show the flow of major transition points (eg, dose escalation to dose expansion, phase 1 to phase 2, single ascending dose to multiple ascending dose) | Results Trial Participants paragraph 1. Figures Figure 1 |
|  |  |  | 3a.3* | Statistical methods or rationale underpinning the trial design | Methods Statistical Analysis paragraph 4 |
|  |  |  | 3a.4* | Starting dose(s) with rationale | Methods Trial Intervention and Procedures paragraph 4 |
|  |  |  | 3a.5* | Range of planned dose levels with rationale | Methods Trial Intervention and Procedures paragraph 4 |
|  |  |  | 3a.6* | Presentation of planned dose levels (eg, as a diagram, table, or infographic), where applicable | Figures Figure 1 |
|  |  |  | 3a.7* | Skipping of dose level(s), if applicable | NA |
|  |  |  | 3a.8* | Planned cohort size(s) (eg, fixed, flexible, adaptive) | Methods Trial Intervention and Procedures paragraph 4 |
|  |  |  | 3a.9* | Dose allocation method within a dose level (including sequence and interval between dosing of participants, eg, sentinel or staggered dosing) | Methods Trial Design and Oversight paragraph 2 |
|  |  |  | 3a.10* | Dose expansion cohort(s), if applicable, with rationale | NA |
|  |  |  | 3a.11* | Criteria for progression to the next part of the trial (eg, phase 1 to phase 2, single ascending dose to multiple ascending dose), where applicable | NA |
|  | 3b | Important changes to methods after trial commencement (such as eligibility criteria), with reasons | 3b† | Important changes to the design or methods after trial commencement (eg, insertion of unplanned additional doses) outside the scope of the prespecified adaptive design features, with reasons | NA |
| Participants | 4a | Eligibility criteria for participants | 4a |  | Methods Trial Participants paragraph 3 |
|  | 4b | Settings and locations where the data were collected | 4b |  | Methods Trial Design and Oversight paragraph 1 |
| Interventions | 5 | The interventions for each group with sufficient details to allow replication, including how and when they were actually administered | 5a† | Interventions for each dose level (within each group) with sufficient details to allow replication, including administration route and schedule showing how and when they were actually administered | Methods Trial Interventions and Procedures paragraph 4 |
|  |  |  | 5b* | Criteria for dose discontinuation, dose modifications, and dosing delays of allocated interventions for a given trial participant (eg, dose change in response to harms, participant request, or improving or worsening disease) | NA |
| Outcomes | 6a | Completely defined prespecified primary and secondary outcome measures, including how and when they were assessed | 6a† | Primary and secondary outcomes, including the specific measurement variable, analysis metric, method of aggregation, and time point for each outcome. Explanation of the clinical relevance of chosen outcomes is strongly recommended. Any other outcomes used to inform prespecified adaptations should be described with the rationale | Methods Primary Outcomes paragraph 2 |
|  | 6b | Any changes to trial outcomes after the trial commenced, with reasons | 6b† | Any unplanned changes to trial outcomes after the trial commenced, with reasons | NA |
| Sample size | 7a | How sample size was determined | 7a† | Estimated number of participants (minimum, maximum, or expected range) needed to address trial objectives and how it was determined, including clinical and statistical assumptions supporting any sample size and operating characteristics | Methods Trial Interventions and Procedures paragraph 4 |
|  | 7b | When applicable, explanation of any interim analyses and stopping guidelines | 7b† | Prespecified interim decision making criteria or rules that guided the trial adaptation process (eg, dosing decision to (de-)escalate); prespecified and actual timing and frequency of interim data reviews and the information to inform trial adaptations | NA |
| Randomization (if applicable) | | | | | |
| Sequence generation | 8a | Method used to generate the random allocation sequence | 8a |  | NA |
|  | 8b | Type of randomisation; details of any restriction (such as blocking and block size) | 8b† | Type of randomisation; details of any restrictions (such as blocking and block size); any prespecified adaptive assignment rules or algorithm leading to adjustments in the allocation ratio, including timing and frequency of updates; any changes to the allocation rule following trial adaptation decisions | NA |
| Allocation concealment mechanism | 9 | Mechanism used to implement the random allocation sequence (such as sequentially numbered containers), describing any steps taken to conceal the sequence until interventions were assigned | 9 |  | NA |
| Implementation | 10 | Who generated the random allocation sequence, who enrolled participants, and who assigned participants to interventions | 10 |  | NA |
| Blinding | 11a | If done, who was blinded after assignment to interventions (eg, participants, care providers, and how | 11a |  | NA |
|  | 11b | If relevant, description of the similarity of interventions | 11b |  | NA |
| Statistical methods | 12a | Statistical methods used to compare groups for primary and secondary outcomes | 12a.1† | Statistical methods for primary and secondary outcomes and any other outcomes used to make prespecified adaptations | Methods Statistical Analysis paragraph 4 |
|  |  |  | 12a.2* | For the implemented adaptive design features, statistical methods used for estimation (eg, safety, dose(s), treatment effects) and to make inferences | NA |
|  | 12b | Methods for additional analyses, such as subgroup analyses and adjusted analyses | 12b† | Statistical methods for additional analyses (eg, subgroup and adjusted analyses, pharmacokinetics or pharmacodynamics, biomarker correlative analyses) | Methods Statistical Analysis paragraph 4 |
|  |  |  | 12c* | Analysis population(s) (eg, evaluable population for dose-finding, safety population) | Methods Statistical Analysis paragraph 4 |
|  |  |  | 12d* | Strategies for handling intercurrent events occurring after treatment initiation (eg, how dosing adjustments were handled) that can affect either the interpretation or the existence of the measurements associated with the clinical question of interest, and any methods to handle missing data | NA |
| **Results** | | | | | |
| Participant flow (a diagram is strongly recommended) | 13a | For each group, the numbers of participants who were randomly assigned, received intended treatment, and were analysed for the primary outcome | 13a† | For each group, the number of participants who were assigned to each dose level at each interim analysis (eg, for dosing decisions), received intended treatment, and were analysed for the primary outcome and, if applicable, any other outcomes used to inform prespecified adaptations | Figures Figure 1 |
|  | 13b | For each group, losses and exclusions after randomisation, together with reasons | 13b† | For each group, losses and exclusions after allocation to each dose level, together with reasons | Results Trial Participants paragraph 1 |
| Recruitment | 14a§ | Dates defining the periods of recruitment and follow-up | 14a§ |  | Methods rial Design and Oversight paragraph 1 |
|  | 14b§ | Why the trial ended or was stopped | 14b§ |  | NA |
|  |  |  | 14c* | Trial adaptation decisions made (including on what basis they were made, and when) in light of the prespecified decision making criteria and observed accrued data | NA |
| Baseline data | 15 | A table showing baseline demographic and clinical characteristics for each group | 15† | Baseline demographic and clinical characteristics across each dose level within each group, where appropriate | Figures and Tables table 1 |
| Numbers analysed | 16 | For each group, number of participants (denominator) included in each analysis and whether the analysis was by original assigned groups | 16† | For each group, the number of participants (denominator) included in each analysis across each dose level, and whether the analysis was by original assigned interventions | NA |
| Outcomes and estimation | 17a | For each primary and secondary outcome, results for each group, and the estimated effect size and its precision (such as 95% confidence interval) | 17a† | For each primary and secondary outcome, results for each dose level within each group, and the estimated effect size and its precision, if applicable | Figures and Tables table 2 |
|  | 17b§ | For binary outcomes, presentation of both absolute and relative effect sizes is recommended | 17b§ |  | NA |
|  |  |  | 17c* | Report interim results used to inform interim decision making such as dose escalation, de-escalation, or staying at the same dose | NA |
| Ancillary analyses | 18 | Results of any other analyses performed, including subgroup analyses and adjusted analyses, distinguishing prespecified from exploratory | 18 |  | NA |
| Harms | 19 | All important harms or unintended effects in each group (for specific guidance, see CONSORT for harms (1)) | 19† | All important harms (eg, adverse events or effects, toxicities) reported by dose level in each group (for specific guidance, see CONSORT for harms (2)) | Figures and Tables table 2 |
| **Discussion** | | | | | |
| Limitations | 20 | Trial limitations, addressing sources of potential bias, imprecision, and, if relevant, multiplicity of analyses | 20 |  | Discussion paragraphs 3 and 4 |
| Generalizability | 21 | Generalisability (external validity, applicability) of the trial findings | 21 |  | NA |
| Interpretation | 22 | Interpretation consistent with results, balancing benefits and harms, and considering other relevant evidence | 22 |  | Discussion paragraph 1 |
| **Other information** | | | | | |
| Registration | 23 | Registration number and name of trial registry | 23 |  | Methods Trial Design and Oversight paragraph 1 |
| Protocol | 24 | Where the full trial protocol can be accessed, if available | 24 |  | NA |
| Funding | 25 | Sources of funding and other support (such as supply of drugs), role of funders | 25 |  | NA |
| Data monitoring |  |  | 26a* | Composition of any decision making or safety review committee or group; summary of its role and reporting structure; statement of whether it is independent from the sponsor and competing interests; and reference to where further details can be found (such as in a charter or protocol) | Methods Trial Design and Oversight paragraphs 1 and 2 |
|  |  |  | 26b* | Description of who had access to interim results and made the interim and final decision to terminate the trial (or part(s) of the trial, eg, end of dose escalation), and measures to safeguard the confidentiality of interim information | NA |
| Dissemination |  |  | 27* | Specify, if applicable, whether and when results (such as safety and/or activity) were reported externally (eg, through scientific presentations, journal publication, or the trial website) while the trial (or part(s) of the trial) was still ongoing | NA |

CONSORT=CONonsolidated Standards Of Reporting Trials; DEFINE=Dose-finding Extension; EPDF=early phase dose-finding.

^ This checklist should be used in conjunction with the CONSORT explanation and elaboration document (3) for important clarifications on the checklist items. Empty items in the CONSORT-DEFINE column indicate no modification from the standard CONSORT item. CONSORT extensions for non-pharmacological treatments and outcomes might also be relevant (4). Note that the term “dose” in the checklist can be considered synonymous and used interchangeably with dosage, or dosing regimen (dose or schedule), or a unit dose.

¤ If the information is not provided in the primary paper, give details of where this information is available. This may include locations such as a published protocol or other published papers (provide citation details) or a website (provide the URL).

* New items that should only be applied in reference to CONSORT-DEFINE.

† Modified items that require reference to both CONSORT and CONSORT-DEFINE.

§ Item wording remains unchanged in reference to CONSORT, but additional CONSORT-DEFINE explanatory text has been provided to clarify additional considerations for early phase dose-finding trials (web appendix 3).
