# Supplementary material for: Safety and tolerability of tegoprubart in patients with amyotrophic lateral sclerosis: A Phase 2A clinical trial
Source: PLoS Med. 2024 Oct 31;21(10):e1004469. doi: 10.1371/journal.pmed.1004469 (PMC11527214; doi:10.1371/journal.pmed.1004469)
Supplement: S1 PROTOCOL — (DOCX) [file pmed.1004469.s002.docx]

**CLINICAL TRIAL PROTOCOL**

**Compound: AT-1501 (tegoprubart)**

**Protocol No: AT-1501-A201**

**Protocol Title:**

**IND Number: 136765**

**Version: 04**

**Date: 08 March 2021**

**1 PROTOCOL SYNOPSIS**

**Product**: AT-1501 1.0, 2.0, 4.0 or 8.0 mg/kg for IV infusion

**Study Title**: A Phase 2a Open-Label, Multi-Center Study to Evaluate the Safety and Tolerability of Multiple Doses of AT-1501 in adults with ALS

**Centers**: This study will be performed at up to twelve centers in the United States and one site in Canada.

**Primary Objective**: To determine the safety and tolerability of IV administration of multiple doses of AT-1501 in adults with ALS.

**Exploratory Objectives**:

- To determine the effect on the ALS Functional Rating Scale – Revised (ALSFRS-R) and spirometry as a result of administration of multiple doses of AT-1501.
- To quantify the effect of treatment on biomarkers of inflammation (e.g. TNFα, IL6, Enrage), neurodegeneration (e.g. NFL & NFH) and other proteins in peripheral blood.
- To understand the immunogenicity profile of AT-1501.
- To correlate biomarkers with AT-1501 levels simulated from study AT-1501-101 PK profiles and plasma concentrations from the current study.

**Study Design**

This is a Phase 2a, multi-center, open label, multiple dose study of AT-1501, a humanized monoclonal antibody antagonist to CD40LG. Approximately 54 adults with ALS will be enrolled into the study in the United States at up to 12 ALS treatment sites.

Four ascending doses of AT-1501 (1.0 mg/kg; 2.0 mg/kg; 4.0 mg/kg and 8.0 mg/kg) will be administered as a 1-hour IV infusion to sequentially enrolling cohorts. The 1.0 mg/kg and 2.0 mg/kg cohorts will consist of 9 participants each and the 4.0 mg/kg and 8.0 mg/kg cohorts will consist of 18 participants each. The participants in each cohort will receive 6 bi-weekly (every other week) infusions of AT-1501 over an 11-week period.

This study consists of a 4 week screening period, an 11 week dosing period in which participants are dosed once every other week, and a 4 week follow-up period. On Day 1 eligible participants will receive their first 1-hour intravenous (IV) infusion of AT-1501 and will be observed for at least 2 hours post infusion before being released from the clinical site. They will return on Weeks 3, 5, 7, 9 and 11 for subsequent infusions and will remain at the clinical site for at least 2 hours for observation and assessments before being released. Participants will be called weekly between dosing visits (Weeks 2, 4, 6, 8, 10 and 12) by a member of the research team to record adverse events (AE) and changes in concomitant medications. Participants will be followed for an additional 4 weeks after their last dose to monitor for safety, with an end of study clinic visit occurring Week 15.

Each participant will be monitored for safety throughout the study. Safety will be assessed by evaluation of AEs, changes in safety laboratory parameters, physical examination including vital signs, ECGs and the Columbia Suicide Severity Rating Scale (C-SSRS). Plasma to measure AT-1501 concentration will be obtained on each dosing day prior to the IV infusion and 2 hours after the start of the infusion. Immunogenicity and exploratory biomarker samples will be taken throughout the study and analyzed at the end of the study. Participant focused clinical outcomes will be assessed at specified timepoints.

The dose limiting toxicity observation period to determine the safety of a dose level is defined as starting with administration of the first dose of AT-1501 to the first participant in a cohort through the administration of the second dose of AT-1501 in the 6th participant in that cohort (See 10.3 for Stopping Rules and Dose Limiting Toxicity).

The Data Monitoring Committee (DMC) will meet following the administration of 6 doses to a minimum of 33% of participants, in each cohort, (3 participants in cohorts 1 and 2 will have received 6 doses for a total of 18 doses, and 6 participants in cohorts 3 and 4 will have received 6 doses for a total of 36 doses) to review safety data and to make dose escalation recommendations to Anelixis.

**Target Population**: Adults with ALS who meet the inclusion/exclusion criteria.

**Safety**: Each participant will be monitored for safety throughout the study. Safety will be assessed by evaluation of AEs, changes in safety laboratory parameters (with special attention to platelet and coagulation values), physical examination including vital signs, ECGs and the C-SSRS.

**Other Outcomes:** Blood samples for AT-1501 plasma concentration, immunogenicity and exploratory biomarker samples will be taken and analyzed at the end of the study. Participant focused clinical outcomes (ALSFRS-R and spirometry) will be assessed at specified timepoints.

**Study Duration**: Participant enrollment, treatment and follow up for all 4 cohorts is estimated to take approximately 18 months. Study duration per participant is approximately 19 weeks: 4 weeks screening, 11 weeks dosing (6 doses every other week) and 4 weeks follow up.

**2 TABLE OF CONTENTS**

[**1** **PROTOCOL SYNOPSIS 4**](#_Toc44082907)

[**2** **TABLE OF CONTENTS 6**](#_Toc44082908)

[2.1 Abbreviations and Definition of Terms 9](#_Toc44082909)

[**3** **INTRODUCTION 10**](#_Toc44082910)

[3.1 Humanized Blocking Antibody to CD40LG 10](#_Toc44082911)

[3.2 Other Experiences with Drugs Targeting CD40LG 10](#_Toc44082912)

[3.3 Preclinical Experience with AT-1501 11](#_Toc44082913)

[3.4 Dose Selection and Rationale 12](#_Toc44082914)

[**4** **OBJECTIVES OF THE TRIAL 14**](#_Toc44082915)

[4.1 Primary Objective 14](#_Toc44082916)

[4.2 Exploratory Objectives 14](#_Toc44082917)

[**5** **INVESTIGATIONAL TRIAL DESIGN 14**](#_Toc44082918)

[5.1 Design 14](#_Toc44082919)

[5.2 Endpoints 15](#_Toc44082921)

[5.2.1 Primary Endpoint 15](#_Toc44082922)

[5.2.2 Exploratory Endpoints 16](#_Toc44082923)

[5.3 Dose Escalation 16](#_Toc44082924)

[5.4 Data Monitoring Committee 17](#_Toc44082925)

[5.5 Concomitant Therapy 17](#_Toc44082926)

[5.6 Excluded Medications 17](#_Toc44082927)

[5.6.1 Concomitant Use of Radicava® (edaravone) and Rilutek® (riluzole) or Tiglutik (riluzole suspension) 18](#_Toc44082928)

[5.7 Study Duration 18](#_Toc44082930)

[**6** **STUDY POPULATION 18**](#_Toc44082931)

[6.1 Number of Participants 18](#_Toc44082933)

[6.2 Participant Screening 18](#_Toc44082934)

[6.3 Inclusion Criteria 19](#_Toc44082935)

[6.4 Exclusion Criteria 19](#_Toc44082936)

[6.5 Handling of Withdrawals 20](#_Toc44082937)

[6.6 Study Drug Discontinuation 20](#_Toc44082938)

[**7** **STUDY PROCEDURES 21**](#_Toc44082939)

[7.1 Screening (Week -4 to Week -1) 21](#_Toc44082941)

[7.2 Dosing Day (Week 1 / Day 1) 22](#_Toc44082942)

[7.3 Phone Call Checks (Weeks 2, 4, 6, 8, 10 and 12; Days 8, 22, 36, 50, 64 and 78 +/- 2 days) 23](#_Toc44082943)

[7.4 Dosing Day (Weeks 3, 5, 7, 9 and 11; Days 15, 29, 43, 57, and 71 +/- 2 days) 23](#_Toc44082944)

[7.5 End of Study (Week 15 +/- 1 week) 24](#_Toc44082945)

[**8** **PROCEDURES AND EVALUATIONS 24**](#_Toc44082946)

[8.1 Assessments of Safety 24](#_Toc44082947)

[8.1.1 Medical History and Physical Examination 24](#_Toc44082948)

[8.1.2 Electrocardiogram 25](#_Toc44082949)

[8.1.3 Columbia Suicide Severity Rating Scale 25](#_Toc44082950)

[8.1.4 Clinical Laboratory 25](#_Toc44082951)

[8.2 Other Assessments 26](#_Toc44082952)

[8.2.1 Clinical Outcome Measures 26](#_Toc44082953)

[8.2.2 Special Assays or Procedures 27](#_Toc44082954)

[**9** **DESCRIPTION OF INVESTIGATIONAL PRODUCT 27**](#_Toc44082955)

[9.1 Sample Vial Label 27](#_Toc44082956)

[9.2 Drug Ordering and Storage 28](#_Toc44082957)

[9.3 Dosage and Administration 28](#_Toc44082958)

[9.4 Drug Accountability 28](#_Toc44082959)

[**10** **ADVERSE EVENTS 29**](#_Toc44082960)

[10.1 Definition of Adverse Events 29](#_Toc44082961)

[10.1.1 Reporting of Adverse Events 29](#_Toc44082962)

[10.1.2 Assessment of Severity of Adverse Event 30](#_Toc44082963)

[10.1.3 Onset of Adverse Events 30](#_Toc44082964)

[10.1.4 Relationship of Adverse Event to Investigational Product 30](#_Toc44082965)

[10.2 Serious Adverse Events 31](#_Toc44082966)

[10.2.1 Definition of a Serious Adverse Event 31](#_Toc44082967)

[10.2.2 Reporting of Serious Adverse Events 31](#_Toc44082968)

[10.3 Stopping Rules and Dose Limiting Toxicity (DLT) 33](#_Toc44082969)

[**11** **STATISTICAL EVALUATION 34**](#_Toc44082970)

[11.1 Justification of Sample Size 34](#_Toc44082971)

[11.2 Definition of Study Analysis Sets 34](#_Toc44082972)

[11.2.1 Safety Analysis Set 34](#_Toc44082973)

[11.2.2 Full Analysis Set 34](#_Toc44082974)

[11.2.3 Evaluable Analysis Set 34](#_Toc44082975)

[11.3 Statistical Analysis 34](#_Toc44082976)

[11.3.1 Study Baseline 34](#_Toc44082977)

[11.3.2 Disposition of Participants 34](#_Toc44082978)

[11.3.3 Demography and Baseline Characteristics 35](#_Toc44082979)

[11.3.4 Safety 35](#_Toc44082980)

[11.3.5 Clinical Outcome Assessments 35](#_Toc44082981)

[11.3.6 Medical History 35](#_Toc44082982)

[11.3.7 Prior and Concomitant Medications 35](#_Toc44082983)

[11.4 Handling of Missing Data 36](#_Toc44082984)

[11.5 Interim Analysis 36](#_Toc44082985)

[11.6 Pharmacokinetic Analyses 36](#_Toc44082986)

[**12** **DATA MANAGEMENT 36**](#_Toc44082987)

[12.1 Participant Data Protection 36](#_Toc44082988)

[12.2 Data Handling 36](#_Toc44082989)

[**13** **ADMINISTRATIVE PROCEDURES 37**](#_Toc44082990)

[13.1 Study Completion or Premature Closure 37](#_Toc44082991)

[13.2 Good Clinical Practice 38](#_Toc44082992)

[13.3 Institutional Review Board 38](#_Toc44082993)

[13.4 Participant Informed Consent 38](#_Toc44082994)

[13.5 Regulatory Affairs 39](#_Toc44082995)

[13.6 Trial Monitoring 39](#_Toc44082996)

[13.7 Trial Audits and Inspections 39](#_Toc44082997)

[13.8 Financing 39](#_Toc44082998)

[13.9 Records Retention 39](#_Toc44082999)

[**14** **CONFIDENTIALITY AND COMMUNICATION OF RESULTS 40**](#_Toc44083000)

[14.1 Statistical and Clinical Reports 40](#_Toc44083001)

[14.2 Regulatory Use of Data 40](#_Toc44083002)

[**15** **REFERENCES 41**](#_Toc44083003)

[**Appendix A** **Schedule of Events 44**](#_Toc44083004)

**2.1 Abbreviations and Definition of Terms**

| **Abbreviation** | **Definition** |
| --- | --- |
| AE | Adverse Event |
| ALS | Amyotrophic Lateral Sclerosis |
| ALSFRS-R | ALS Functional Rating Scale – Revised |
| ALT | Alanine aminotransferase (alanine transaminase) |
| AST | Aspartate aminotransferase (aspartate transaminase) |
| CD40LG | CD40 Ligand |
| CBC | Complete Blood Count |
| CRF | Case Report Form |
| CRO | Contract Research Organization |
| C-SSRS | Columbia Suicide Severity Rating Scale |
| CTCAE | Common Terminology Criteria for Adverse Events v 4.03 |
| CV | Curriculum Vitae |
| ECG | Electrocardiogram |
| FAS | Full Analysis Set |
| GMP | Good Manufacturing Practice |
| ICH | International Conference of Harmonization |
| IRB | Institutional Review Board |
| ITT | Intent-to-Treat |
| IU | International unit |
| kg | kilogram |
| MedDRA | Medical Dictionary for Regulatory Activities |
| mL | milliliter |
| MR1 | A hamster antibody targeted against murine CD40LG. |
| NHP | Non-human primate |
| PP | Per-Protocol |
| PT | MedDRA Preferred Term |
| SAE | Serious Adverse Event |
| SAP | Statistical Analysis Plan |
| Sec | Seconds |
| SDV | Source Data Verification |
| SOA | Schedule of Activities |
| SOC | MedDRA System Organ Class |
| SOP | Standard Operating Procedure |
| SPC | Summary of Product Characteristics |
| SVC | Slow Vital Capacity |
| TE | Thromboembolism |
| TEAE | Treatment-Emergent Adverse Event |
| WBC | White Blood Cell Count |

All dimensional units are in standard SI units

**3 INTRODUCTION**

Amyotrophic Lateral Sclerosis (ALS) is a fatal, degenerative disorder of motor neurons that results in progressive wasting and paralysis of voluntary muscles. There is mounting evidence that the disease progression is mediated in part by immune activation. Prior research aimed at non-specific immunomodulatory therapies targeting the immune system broadly have not identified successful therapies for ALS. Antibodies blocking CD40LG have been shown to successfully reduce the activation of circulating lymphocytes, monocytes, macrophages, and other antigen presenting cells (see Section 0).

This multiple ascending dose protocol will study the safety, tolerability, and plasma concentrations of AT-1501 in adults with ALS.

A First-in-Human single ascending dose study with doses of 0.5 mg/kg; 1.0 mg/kg; 2.0 mg/kg; 4.0 mg/kg and 8 mg/kg was successfully carried out with no safety or tolerability concerns. The safety data and pharmacokinetics of this initial study is available in the Investigator’s Brochure.

**3.1 Humanized Blocking Antibody to CD40LG**

Preclinical testing in a murine model of ALS has demonstrated statistically significant slowing of the disease course in animals treated with MR1, a hamster antibody targeted against murine CD40LG. [Lincecum 2010]. Furthermore, analysis reveals decreased immune activation in treated animals, with less macrophage invasion of nerves in the periphery and less gliosis in the central nervous system. Based on this evidence, a humanized blocking antibody to CD40LG, AT-1501, was tested in healthy adults (0.5, 1.0, 2.0, 4.0 and 8.0 mg/kg) as well as adults with ALS in a single ascending dose study. (Three adults with ALS received 1.0 mg/kg.)

**3.2 Other Experiences with Drugs Targeting CD40LG**

Antibodies blocking CD40LG signaling have shown efficacy in preclinical models of Systemic Lupus Erythematosus (SLE) [Mohan 1995; Early 1996; Daikh 1997; Kalled 1998; Wang 2002], Multiple sclerosis (MS) [Gerritse 1996; Samoilova 1997; Howard 1999; Schaub 1999; Laman 2002], graft versus host disease [Durie 1994; Parker 1995; Larsen 1996], inflammation mediated retinopathies and Alzheimer’s Disease [Tan 2001; Town 2001; Tan 2002]. However, clinical trials for SLE using anti CD40LG antibodies (Idec-131, BG9588) exhibited significant platelet aggregation, leading to thromboembolisms and acute toxicity by binding to the normally high expression of CD40LG on platelets [Gobburu 1998; Kawai 2000; Kalunian 2002; Boumpas 2003; Kuwana 2004]. Yet in these Phase II SLE studies, antibodies blocking CD40LG function demonstrated clear target engagement and treatment positively impacted both clinical outcome measures and inflammatory biomarkers in SLE patients.

Engineering of human antibodies targeting CD40LG have proven that platelet aggregation and its associated toxicity is due to Fc effector function [Shock 2015; Kim 2017]. Antibodies engineered to abrogate Fcµ receptor interactions or pegylated FAbs (CDP7657) that lack the Fc domain entirely, do not promote platelet aggregation and show improved safety profiles are now in clinical development for SLE by UCB Pharma [Wakefield 2010; Chamberlain 2017].

AT-1501 was designed to mitigate the risk of thrombotic events seen with an earlier anti-CD40LG antibody by introducing structural modifications that do not alter binding of AT-1501 to CD40LG but reduce antibody dependent cellular cytotoxicity (ADCC), complement activation and platelet activation associated with Fcγ receptor binding [Saxena 2016]. The preclinical data support a low risk of thromboembolic (TE) events in susceptible animal models.

To our knowledge, AT-1501 is the only biologic targeting CD40LG in development for treatment of neurodegenerative conditions. AT-1501 is also a very different product candidate compared to CDP7657. AT-1501 is a full IgG1 antibody with very different affinity, pharmacokinetics, and biodistribution compared to CDP7657, a pegylated FAB.

**3.3 Preclinical Experience with AT-1501**

The CD40LG sequence in several non-human primate (NHP) species, including rhesus macaques, baboons, and cynomolgus macaques, are identical to each other and differ from the human sequence by only five residues, with one of those residues residing on the extracellular portion of the molecule that is accessible for antibody binding (<https://blast.ncbi.nlm.nih.gov/Blast.cgi>). AT-1501 binds to human, rhesus macaque, and cynomolgus CD40LG.

The thromboembolic (TE) events associated with the clinical development of the reference antibody, Hu5c8 have been reported in cynomolgus and rhesus monkeys in renal allograft studies [Kanmaz 2004; Koyama 2004; Schuler 2004]. However, rhesus monkey appears to be more sensitive with TE events noted at doses as low as 5 mg/kg; while doses in normal cynomolgus monkeys up to 50 mg/kg did not result in TE events [Gobburu 1998; Nadler 2014; Shock 2015]. Since TE events with Hu5c8 were not reported in humans at doses <20 mg/kg [Kalunian 2002; Kuwana 2004], the rhesus monkey may be more sensitive to the TE events, and therefore represents an appropriately sensitive model for detecting the potential for TE events by anti-CD4LG IgG1 antibodies in humans. AT-1501 binds to both human and rhesus macaque CD40LG, indicating that this NHP species is appropriate for studying the safety of AT-1501 (Anelixis internal data).

**3.4 Dose Selection and Rationale**

Based on the evidence above, a humanized blocking antibody to CD40LG, AT-1501, was tested in healthy adults and adults with ALS in a randomized, placebo-controlled, double-blind, dose escalation trial. A total of 32 adults; 28 healthy subjects and 4 adults with ALS were enrolled into Cohorts 1-6 (Table 1). Each subject received a single dose AT-1501 or placebo, on Study Day 1, and was followed for safety for 6 weeks (43 days). The primary objective of the study was to determine the safety and tolerability of IV administration of a single dose of AT-1501 in healthy adult volunteers and of one dose in adults with ALS; the secondary objective was to determine the plasma pharmacokinetics of AT-1501 following single ascending doses of AT-1501.

Table 1 AT-1501-101 Dosing Scheme

| Cohort | AT-1501 Dose (mg/kg) | Population | No. Active Subjects | No. Placebo Subjects |
| --- | --- | --- | --- | --- |
| 1 | 0.5 | HS | 6 | 2 |
| 2 | 1.0 | HS | 3 | 1 |
| 3 | 1.0 | ALS | 3 | 1 |
| 4 | 2.0 | HS | 3 | 1 |
| 5 | 4.0 | HS | 3 | 1 |
| 6 | 8.0 | HS | 6 | 2 |

HS = Healthy subject

ALS = Subjects with ALS

The doses of AT-1501 administered in the Phase 1 study (0.5, 1, 2, 4, and 8 mg/kg) demonstrated a safety profile comparable to placebo, were well tolerated, and warrant further investigation in adults with ALS. There were no deaths, SAEs, or TEAEs that led to study or study drug discontinuation. There were no clinically significant laboratory results, vital sign assessments, ECG assessments, or physical examination findings. No subject expressed suicidal ideation. Subjects with ALS who were treated with AT-1501 had a similar safety profile as healthy subjects treated with AT-1501 and subjects treated with placebo.

Mean plasma concentrations of AT-1501 increased in a manner that was dose proportional over the dose range tested. The PK parameters in adults with ALS were consistent with those from healthy volunteers at the 1.0 mg/kg dose level. The ADA antibody titers were low, and because circulating levels of AT-1501 were as predicted for all cohorts, these data suggest that the ADA antibody responses were not neutralizing.

The Phase 2a clinical study will test the safety and tolerability of multiple ascending doses (1.0, 2.0, 4.0, and 8.0 mg/kg) of AT-1501 in adults with ALS and includes exploratory assessment of biomarkers of inflammation and neurodegeneration. Nine participants in each of the lower dose cohorts (1 and 2 mg/kg) will allow for initial review of safety at multiple doses while eighteen participants in the higher cohorts (4 and 8 mg/kg) allows for reduced variability to assess exploratory biomarkers as well as safety.

**4 OBJECTIVES OF THE TRIAL**

**4.1 Primary Objective**

To determine the safety and tolerability of IV administration of multiple doses of AT-1501 (planned 1.0, 2.0, 4.0 and 8.0 mg/kg).

**4.2 Exploratory Objectives**

- To determine the effect on ALSFRS-R and spirometry as a result of administration of multiple doses of AT-1501.
- To quantify the effect of treatment on biomarkers of inflammation (e.g. TNFα, IL6, Enrage), neurodegeneration (e.g. NFL & NFH) and other proteins in peripheral blood.
- To understand the immunogenicity profile of AT-1501.
- To correlate biomarkers with AT-1501 levels simulated from study AT-1501 PK profiles and plasma concentrations from the current study.

**5 INVESTIGATIONAL TRIAL DESIGN**

**5.1 Design**

This is a Phase 2a, multi-center, open label, multiple dose study of AT-1501, a humanized monoclonal antibody antagonist to CD40LG. Approximately 54 adults with ALS will be enrolled into the study in the United States at up to 12 ALS treatment sites in the United States and 1 site in Canada.

Four ascending doses of AT-1501 (1.0 mg/kg; 2.0 mg/kg,4.0 mg/kg and 8.0 mg/kg) will be administered as a 1-hour IV infusion to sequentially enrolling cohorts. The 1.0 mg/kg and 2.0 mg/kg cohorts will consist of 9 participants each and the 4.0 mg/kg and 8.0 mg/kg cohorts will consist of 18 participants each. The participants in each cohort will receive 6 bi-weekly (every other week) infusions of AT-1501 over an 11-week period.

The primary objective of the study is to assess safety and tolerability. The exploratory goal to correlate biomarkers of drug response and plasma concentrations of AT-1501 will facilitate dose selection for subsequent clinical trials. While the trial is underpowered to assess efficacy with clinical endpoints, the exploratory endpoints using two participant focused outcomes – ALS Functional Rating Scale-R (ALSFRS-R) and spirometry may demonstrate trends in functional improvement within participants over the short duration of the study, particularly in higher dose cohorts with 18 participants each. In order to comply with differing state mandates aimed at controlling the spread of COVID-19 some hospitals and clinics may limit or prohibit spirometry testing. Therefore, the use of the ALSFRS-R will be utilized for inclusion of all participants into the study and to provide a baseline for the exploratory endpoint. Spirometry data will be collected at baseline and Weeks 5, 9 and 11, and at the End of Study visit provided the site has the ability and permission to safely conduct the test.

This study consists of a 4-week screening period, an 11-week dosing period in which participants are dosed once every other week, and a 4 week follow-up period. On Day 1 eligible participants will receive their first 1-hour intravenous (IV) infusion of AT-1501 and will be observed for at least 2 hours post infusion before being released. They will return for clinical visits on Weeks 3, 5, 7, 9 and 11 for subsequent infusions and will remain at the clinical site for at least 2 hours for observation and assessments before being released. Participants will be called weekly between dosing visits (Weeks 2, 4, 6, 8, 10 and 12) by a member of the research team to record adverse events (AE) and changes in concomitant medications. Participants will be followed for an additional 4 weeks after their last dose to monitor for safety, with an end of study clinic visit occurring Week 15.


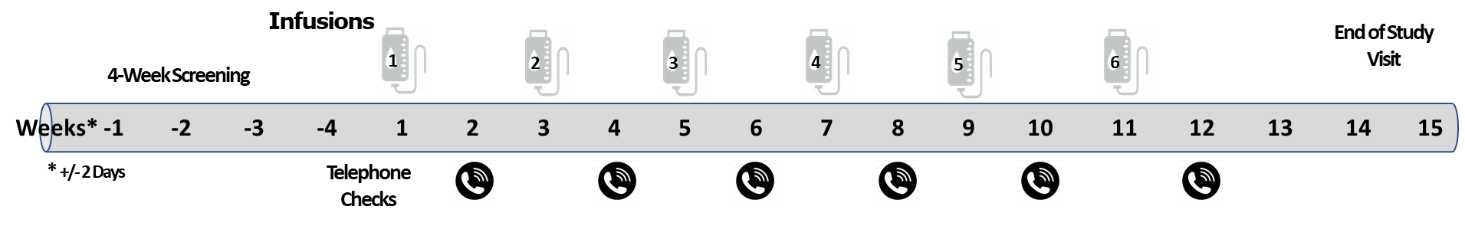


Each participant will be monitored for safety throughout the study. Safety will be assessed by evaluation of AEs, changes in safety laboratory parameters, physical examination including vital signs, ECGs and the Columbia Suicide Severity Rating Scale (C-SSRS). Plasma to measure AT-1501 concentration will be obtained on each dosing day prior to the IV infusion and 2 hours after the start of the infusion. Immunogenicity and exploratory biomarker samples will be taken throughout the study and analyzed at the end of the study. Participant focused clinical outcomes will be assessed at specified timepoints.

The dose limiting toxicity observation period to determine the safety of a dose level is defined as starting with administration of the first dose of AT-1501 to the first participant in a cohort through the administration of the second dose of AT-1501 in the 6th participant in that cohort (See 10.3 for Stopping Rules and Dose Limiting Toxicity).

The Data Monitoring Committee (DMC) will meet following the administration of 6 doses to a minimum of 33% of participants, in each cohort to review safety data and to make dose escalation recommendations to Anelixis. The meetings for dosing cohorts 1 and 2 will be held after 3 participants have received 6 doses, for a total of 18 doses. The meetings for dosing cohorts 3 and 4 will be held after 6 participants have received 6 doses, for a total of 36 doses.

**5.2 Endpoints**

**5.2.1 Primary Endpoint**

Safety and tolerability variables include incidence of adverse events (AEs), changes in physical examination, vital signs, ECG, laboratory parameters and C-SSRS.

**5.2.2 Exploratory Endpoints**

Exploratory clinical outcome measures include ALSFRS-Revised and spirometry (when available).

Biomarkers of inflammation (e.g. TNFα, IL6 and Enrage), neurodegeneration (e.g. NFL and NFH) and other proteins in peripheral blood.

Measurement of AT-1501 plasma concentration and potential formation of antibodies directed at AT-1501.

**5.3 Dose Escalation**

Participants will be enrolled into 1 of 4 sequentially enrolling cohorts (1.0 mg/kg; 2.0 mg/kg; 4.0 mg/kg; and 8.0 mg/kg). Nine participants will be enrolled into each of the 1.0 mg/kg and 2.0 mg/kg cohorts and 18 participants will be enrolled into each of the 4.0 mg/kg and 8.0 mg/kg cohorts. Dose escalation decisions will be made by Anelixis with input from the DMC (See 5.4 Data Monitoring Committee) and Investigators. After the decision has been made to continue dosing a cohort the next dose cohort will be allowed to start, once the last participant enrolled to the current cohort has received their first dose. This process will be followed for each cohort.


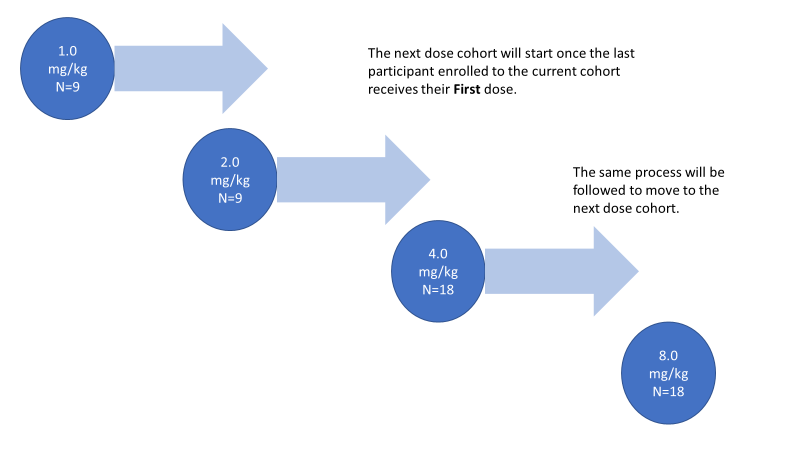


The planned dose escalation scheme may be amended based on review of the emerging data. Additional cohorts may be added, and smaller cohort sizes may be implemented based on safety data and AT-1501 serum concentration results.

**5.4 Data Monitoring Committee**

A DMC will be established and will be comprised of 2 physicians (with ALS experience and no other involvement in the trial) and a statistician.

The committee will meet following the administration of 6 doses to a minimum of 33% of participants, in each cohort. The meetings for dosing cohorts 1 and 2 will be held after 3 participants have received 6 doses, for a total of 18 doses. The meetings for dosing cohorts 3 and 4 will be held after 6 participants have received 6 doses, for a total of 36 doses. The review will include all the available safety and laboratory data, including but not limited to platelet levels and coagulation data. AT-1501 plasma concentration data may be reviewed depending on data availability. The committee may recommend a revision of the dose escalation scheme based on review of the emerging data.

The DMC is responsible for making a recommendation to continue enrolling at the current concentration, escalate to the next planned dose, recommend changes in study conduct, or stop further dosing in the study. Specific responsibilities of the DMC are detailed in the DMC Charter including meeting schedules, documentation requirements, and data review. The DMC may meet on an ad hoc basis should an emergent safety issue be identified (see Section 10.3).

**5.5 Concomitant Therapy**

Generally, concomitant medications can be added, changed or discontinued as medically necessary. Appropriate documentation for all forms of pre-medications, supportive care, and concomitant medications will be captured in the source documents. This includes any immunizations or vaccinations received for the duration of the study.

**5.6 Excluded Medications**

The following medications are exclusionary:

- Systemic administration of corticosteroids (PO/IV/IM) at a dose equivalent to 20 mg/day of prednisone for more than 14 consecutive days within 90 days prior to screening,
- Administration of anti-neoplastic and/or immunomodulating agents (e.g. tumor necrosis factor [TNF] α antagonists or anti-B cell antibodies) or radiotherapy within 1 year prior to screening,
- Cannabinoids unless prescribed or recommended by a physician,
- Any use of experimental therapies,
- Menopausal hormone replacement therapies containing estrogen therapy alone or a combination of estrogen and progesterone agent (E+P),
- Any prior exposure to stem cell and gene therapy (see Section 0 - Exclusion Criteria).

Concomitant medications that are approved drugs or vitamin therapies which the participant may be taking off label are not necessarily exclusionary, recognizing that adults with ALS often take supplemental agents for symptom relief.

**5.6.1 Concomitant Use of Radicava® (edaravone) and Rilutek® (riluzole) or** **Tiglutik (riluzole suspension)**

- Edaravone and riluzole are allowed if the participant has been receiving a stable dose of edaravone for at least 3 months and/or riluzole for at least 1 month prior to Screening.
- Discontinuation of edaravone or riluzole must have occurred at least 3 months for edaravone and 1 month for riluzole prior to Screening for study eligibility.
- The participant and treating physician must anticipate continuing the current treatment regimen with edaravone or riluzole for the duration of the trial.

**5.7 Study Duration**

Study enrollment and follow-up may take up to 18 months from first patient first visit to last patient last visit. Each participant will have a study duration of up to 15 weeks from first dosing to the end of study visit. There is a screening period of up to 4 weeks.

**6 STUDY POPULATION**

Participants will be recruited from neurology practices and through a referral network at facilities treating patients with ALS. Each participating clinical site is evaluated with respect to its ability to enroll the necessary study participants. Strategies for recruitment and retention are discussed during routine monitoring visits. Men and women meeting all the inclusion criteria and none of the exclusion criteria will be allowed to participate.

**6.1 Number of Participants**

The number of participants to be enrolled into the study is approximately 54 adults with ALS.

**6.2 Participant Screening**

The purpose of Screening Visit 1 is to thoroughly inform, both verbally and in writing, the potential participant about the study and allow him/her the opportunity to ask questions. The potential participant will then sign and date the informed consent form and screening procedures can be performed. Once the consent form is signed the participant is considered enrolled into the study and any serious adverse events that occur will be captured from the date of consent until the end of study participation.

**6.3 Inclusion Criteria**

1. Men and women 18 years or older.
2. ALS diagnosed as possible, laboratory supported probable, probable, or definite as defined by revised El Escorial criteria.
3. ALSFRS-R Aggregate score of 35 or greater, and an aggregate score of 9 or greater at Screening for domains 10 Dyspnea, 11 Orthopnea and 12 Respiratory Insufficiency.
4. No more than 24 months from diagnosis.
5. Women of childbearing potential may be enrolled if a pregnancy test is negative, and they agree to the use of 2 forms of contraception from screening to the end of the study. Males must agree to use 2 forms of contraception from screening to the end of the study if their partners are of childbearing potential.

Acceptable methods of birth control which must be used together are:

1. Oral contraceptive and condom (combination oral contraceptives containing the second-generation progestin (levonorgestrel) and <30 µg of estrogen should be utilized),
2. IUD and condom,
3. Diaphragm with spermicide and condom.

d. True abstinence is also an acceptable form of birth control

1. No active suicidal ideation with a plan, or behaviors which may be indicative of a participant’s intent to complete suicide as determined by the completion of the C-SSRS.
2. Able and willing to give informed consent, follow trial procedures, and make multiple clinical site visits.

**6.4 Exclusion Criteria**

1. Any other central or peripheral nervous system disease that may interfere with the evaluation of ALS or its progression.
2. Presence of a tracheostomy, or use of permanent assistive ventilation (ventilatory support for 23 hours per day or more).
3. Previous exposure to AT-1501.
4. History of malignancy within the previous 5 years, except for localized non-melanoma skin cancers.
5. Women who are pregnant, expecting to get pregnant or who are breast feeding.
6. Abnormal function of the immune system resulting from:

- Clinical conditions affecting the immune system (e.g. HIV infection, agammaglobulinemia),
- Systemic administration of corticosteroids (PO/IV/IM) at a dose equivalent to 20 mg/day of prednisone for more than 14 consecutive days within 90 days prior to screening,
- Administration of anti-neoplastic and/or immunomodulating agents (e.g. TNF α antagonists or anti-B cell antibodies) or radiotherapy within 1 year prior to screening.

1. Recipient of Stem Cell or Gene Therapy.
2. Serum AST or ALT value greater than 3 times the ULN.
3. Serum creatinine value greater than 1.5 times the ULN.
4. Positive test for Hepatitis B surface antigen, Hepatitis C antibody, or HIV.
5. History of deep venous thrombosis or pulmonary embolism.
6. Exposure to any other experimental or investigational agent within 30 days or 5 half-lives; whichever is longer.
7. History of active substance abuse within the past 2 years;
8. History of stroke, poorly controlled or significant cardiovascular disease, diabetes or any other acute or chronic medical condition that would unreasonably increase the risks of the study procedures, as determined from an interview with the participant and/or review of medical records.
9. Any other medical condition such as COVID-19, medication, or pre-planned medical/surgical procedure that, in the opinion of the Investigator, would compromise the safety of patients or the integrity of study results.

**6.5 Handling of Withdrawals**

Participants are free to withdraw their consent to the trial at any time without providing reason(s) for withdrawal and without prejudice to further treatment.

It is important to collect safety data on any participant who withdraws consent from the study before their last scheduled visit (End of Study Visit – Week 15). If early withdrawal occurs the clinical site staff is expected to contact the participant for follow-up by both telephone and mail. A minimum of three attempts at contact should be documented, the last with a certified letter, if necessary.

If early withdrawal of consent occurs, the participant should be asked to return for their next scheduled visit (e.g., next study visit or phone assessment) to complete an end-of-study evaluation (Section 7.5). At a minimum the participant should be questioned over the telephone about any changes in clinical condition since their last visit, the status of any ongoing adverse events. The aim is to record data in the same way as for participants who complete the trial.

Patients who withdraw or are withdrawn prior to receiving at least 4 doses of AT-1501 will be replaced within the same dosing cohort that was not completed (i.e. a patient withdrawn from the 1 mg/kg dosing cohort may be replaced with another individual who will receive 1 mg/kg).

**6.6 Study Drug Discontinuation**

Participants may be discontinued from receiving study drug for any of the following reasons:

- Lack of cooperation with study requirements;
- Intercurrent illness, that in the opinion of the Investigator, would unreasonably increase the risks of the study procedures, or otherwise compromise the safety of the participant or the integrity of study results;
- Participant refuses further treatment with AT-1501;
- Actions taken to control the -spread of COVID-19 (e.g. no access to study drug)
- Active COVID-19 infection
- Participant Withdraws consent to the trial;
- Stopping rules are met;
- At the direction of Anelixis;
- At the direction of the Investigator.

The effects of AT-1501 have not been studied in patients that have been diagnosed with COVID-19. It is unknown whether taking the investigational drug will increase or decrease the risk of getting COVID-19 or the effect it may have on the clinical course of a participant who develops COVID-19. Potential participants for the study will be tested for COVID-19 prior to entering the study. If a participant tests positive they will not be allowed to participate in the study. If a participant is diagnosed with COVID-19 while receiving treatment in this study, the administration of the investigational drug will be stopped, and appropriate medical care and follow-up will be done. The participant will continue to be monitored for safety.

All participants that withdraw from treatment with AT-1501 for any reason should continue to be followed until the end of study.

If at any time during the study a participant experiences an adverse event the Investigator believes is severe enough to preclude further AT-1501 administration or the stopping rules are met the participant will be discontinued from study drug treatment and will receive medical treatment as determined by the Investigator. The participant will be followed until the event resolves or until the Investigator determines the AE has reached a stable outcome or is no longer clinically significant. Regardless, each participant will be followed for safety for at least 4 weeks from the last AT-1501 infusion. A final visit will be completed according to the end-of-study evaluation (Section 7.6).

**7 STUDY PROCEDURES**

The assessments to be performed during the study are outlined below. The first day the participant receives AT-1501 is considered “Day 1”. Refer to Appendix A Time and Events Schedule.

**7.1 Screening (Week -4 to Week -1)**

The Screening Visit is performed to inform the patient about the study and check eligibility for participation. No treatment or study related procedures will be initiated before written informed consent has been obtained. Results from the screening visit and procedures conducted as part of routine standard of care are used to determine study eligibility.

- Record medical history and demographics
- Record concomitant medications
- Complete physical exam including weight and height
- Vital signs after 5 minutes in a supine or seated position (blood pressure, heart rate, temperature, respiration rate)
- ECG
- Blood samples for serum chemistry, CBC with differential and platelet count, coagulation tests, Serum pregnancy test (women of child-bearing potential), Hepatitis B surface antigen, Hepatitis C antibody, and HIV
- COVID-19 testing to be done no later than 1 week prior to the first infusion.
- Urinalysis
- Blood samples for baseline biomarkers
- Administration of the ALSFRS-R
- Slow Vital Capacity (SVC), if available
- Administration of the Columbia Suicide Severity Rating Scale (C-SSRS)

**7.2 Dosing Day (Week 1 / Day 1)**

Participants who have signed informed consent and have met all the inclusion criteria and none of the exclusion criteria will continue to Day 1.

**Pre-Dose**

- Review concomitant medications
- Symptom directed physical exam including weight
- Vital signs after 5 minutes in a supine or seated position (blood pressure, heart rate, temperature, respiration rate)
- Blood and urine samples for serum chemistry, CBC with differential and platelet count, coagulation tests, and urinalysis
- Blood sample for AT-1501 concentration, immunogenicity, and biomarkers
- Urine test for pregnancy
- Clinical outcome assessments including ALSFRS-R and spirometry (if available)
- Administration of C-SSRS

**Dose of AT-1501**

1-hour infusion

- Vital signs after 5 minutes in a supine or seated position taken at 15 minutes into the infusion (blood pressure, heart rate, temperature, respiration rate)
- AE assessment

**Post Dose**

- Assessments performed post-infusion are timed from the end of the 1-hour infusion, not the IV-line flush.
- Vital signs after 5 minutes in a supine or seated position (blood pressure, heart rate, temperature, respiration rate) at 1 and 2 hours post-infusion
- ECG 2 hours post-infusion
- Blood samples for AT-1501 concentration 2 hours after the start of the infusion
- AE assessments

**7.3 Phone Call Checks (Weeks 2, 4, 6, 8, 10 and 12; Days 8, 22, 36, 50, 64 and 78 + 2 days)**

- AE assessment
- Record concomitant medications

**7.4 Dosing Day (Weeks 3, 5, 7, 9 and 11; Days 15, 29, 43, 57, and 71 + 2 days)**

**Pre-Dose**

- Symptom directed physical exam (including examination of the injection site and weight)
- Vital signs after 5 minutes in a supine or seated position (blood pressure, heart rate, temperature, respiration rate)
- Record concomitant medications
- Blood and urine samples for serum chemistry, CBC with differential and platelet count, coagulation tests, and urinalysis
- Blood sample for AT-1501 concentration
- Blood samples for immunogenicity (Weeks 3 & 5 only)
- Blood sample for biomarkers
- Administration of C-SSRS
- Administration of ALSFRS-R
- Spirometry (Weeks 5, 9 and 11 only), if available
- AE assessment

**Dose**

- 1-hour infusion
- Vital signs after 5 minutes in a supine or seated position and then taken again at 15 minutes into the infusion (blood pressure, heart rate, temperature, respiration rate)
- AE assessment

**Post-Dose**

- Assessments performed post-infusion are timed from the end of the 1-hour infusion, not the IV-line flush.
- Vital signs after 5 minutes in a seated position (blood pressure, heart rate, temperature, respiration rate) at 1 and 2 hours post-infusion
- Blood sample for AT-1501 concentration 2 hours after the start of the infusion
- ECG taken 2 hours post-infusion
- AE Assessment

**7.5 End of Study (Week 15 + 1 week)**

- Symptom directed physical exam (including examination of the injection site and weight)
- Vital signs after 5 minutes in a supine or seated position (blood pressure, heart rate, temperature, respiration rate)
- Blood and urine sample for serum chemistry, CBC with differential and platelet count, coagulation tests, and urinalysis
- Blood sample for AT-1501 concentration, immunogenicity and biomarkers
- ECG
- Clinical outcome assessments including ALSFRS-R and spirometry (if available)
- Administration of C-SSRS
- Record concomitant medications
- AE assessment

**8 PROCEDURES AND EVALUATIONS**

**8.1 Assessments of Safety**

**8.1.1 Medical History and Physical Examination**

A medical history is obtained through review of the participant’s medical records and personal interview in order to assess eligibility. Assessment of eligibility should include a full review of all concomitant medications and relevant vaccinations or immunizations.

A complete physical examination is performed at the screening visit as well as a COVID-19 test to be conducted no later than a week prior to the first infusion. The complete physical examination will include measurement of height, weight and vital signs. A symptom-directed physical examination (including examination of the injection site and weight) and vital signs is performed pre-dose on the days of dosing. Vital signs include heart rate, respiratory rate, blood pressure (after being seated/supine for at least 5 minutes) and temperature.

A weight change of 10% or greater from the previous measurement will warrant a recalculation of the AT-1501 dose.

**8.1.2 Electrocardiogram**

A 12-lead ECG will be obtained locally by a qualified technician and interpreted locally by a qualified physician. ECGs will be obtained at Screening, post-infusion on dosing days and at the End of Study Visit. Post-infusion ECGs should be read on the day they are taken.

**8.1.3 Columbia Suicide Severity Rating Scale**

The Columbia Suicide Severity Rating Scale, or C-SSRS, will be administered at Screening, pre-dose each dosing day and End of Study, by a certified individual. The scale will be used as a prospective assessment for active suicidal ideation with a plan and to identify specific behaviors which may be indicative of a participant’s intent to complete suicide.

In order to ensure inter-rater reliability raters will be trained centrally according to a standardized protocol.

**8.1.4 Clinical Laboratory**

Blood samples do not need to be drawn fasting. Blood samples for routine safety labs will be drawn locally, processed and shipped to the following central laboratory for testing. Please refer to the Laboratory Manual for additional details.

CENETRON CENTRAL LABORATORIES
Corporate and Clinical Trials
2111 W. Braker Lane Suite 300
Austin, Texas 78758
Telephone: (888) 834-6632
Fax: (512) 439-5006
Email: [info@cenetron.com](mailto:info@cenetron.com)

Tests to be done may include:

**Serum Chemistry:** sodium, potassium, chloride, magnesium, bilirubin, alkaline phosphatase (ALP), alanine aminotransaminase (ALT), aspartate aminotransferase (AST), Lactate dehydrogenase (LDH), urea, creatinine, phosphorus, calcium, glucose, albumin, total protein.

**Hematology:** CBC (including hematocrit and hemoglobin) with, differential and platelet count, coagulation tests (PT, PTT, INR), fibrinogen, neutrophils, lymphocytes, monocytes, eosinophils, basophils.

**Serum Pregnancy Test** must be performed for women of child-bearing potential. Initial screening results will be used to determine if the participant is eligible for protocol enrollment.

**Blood Tests** for HIV, Hepatitis B surface antigen and Hepatitis C antibody

**Urinalysis:** blood, bilirubin, glucose, ketones, leukocytes, nitrites, pH, protein, specific gravity, urobilinogen, microscopic (bacteria, cast, crystals epithelial cells, mucus, RBC, WBC)

See Appendix A for the Time and Events Schedule and the Laboratory Manual for additional details regarding collection, processing and shipping of samples.

**8.2 Other Assessments**

**8.2.1 Clinical Outcome Measures**

Two participant focused outcomes will be explored to assess functional improvement within a patient over the short duration of the trial: the ALS Functional Rating Scale-Revised (ALSFRS-R) and spirometry. The ALSFRS-R will be administered at Screening and pre dose on all dosing days, and at the End of Study visit.

In order to comply with differing state mandates aimed at controlling the spread of COVID-19 some hospitals and clinics may limit or prohibit spirometry testing. Spirometry data will be collected at baseline and Weeks 5, 9 and 11, and at the End of Study visit provided the site has the ability and permission to safely conduct the test.

In order to ensure inter-rater reliability raters will be trained centrally according to a standardized protocol.

**8.2.2 Special Assays or Procedures**

Blood samples for special assays will be drawn locally, processed and shipped to a central laboratory for testing. Samples will be assayed using a validated analytical method. See Appendix A for the Time and Events Schedule and the Laboratory Manual for additional details regarding collection, processing and shipping of samples.

Celerion
621 Rose Street, Lincoln, NE 68502
telephone: 1 531-207-1327

Email: Leslie.engelman@celerion.com

**8.2.2.1 Biomarkers**

Blood samples for biomarker analysis will be taken at Screening, pre- dose on all dosing days and at End of Study. Biomarkers of inflammation (e.g. TNFα, IL 6), neurodegeneration (e.g. NFL and NFH) and other proteins in peripheral blood will be measured. Additional biomarkers with correlation to the disease may also be measured.

**8.2.2.2 AT-1501 Concentration**

Blood samples will be drawn prior to each 1-hour infusion, and 2 hours after the start of the infusion on all dosing days, and at End of Study.

**8.2.2.3 Immunogenicity**

Blood samples will be drawn for anti-AT-1501 antibody detection prior to each 1-hour infusion, on Weeks 1, 3 and 5, and End of Study.

**9 DESCRIPTION OF INVESTIGATIONAL PRODUCT**

AT-1501 is supplied as a lyophilized powder in a single use 6mL glass vial with a rubber stopper and flip-off aluminum seal. Each vial contains 40 mg AT-1501 and is intended for single use only. There is no preservative in the AT-1501 drug product. Reconstitution with 2.2 mL sterile water for injection (sWFI) yields 20 mg/mL AT-1501 solution containing 25 mM Histidine, 225 mM Sucrose, 0.02% (w/v) polysorbate 20 (pH 6.5).

The drug is to be reconstituted in the pharmacy. The reconstituted liquid is clear and will be injected into 100 mL of saline solution for infusion. See the Pharmacy Manual for instructions on reconstitution, storage and preparing the infusion bag.

**9.1 Sample Vial Label**

Labelling will be performed in English at the drug distribution center. See sample label below:

| **AT-1501 for IV Use Only Anelixis Therapeutics**  **Single Use Vial** Lot #036-001-001  **Prior to Reconstitution: Store at 2 to 8°C**  **See Pharmacy Manual for reconstitution, use and storage instructions**  **20 mg/mL after reconstitution**  **Caution: New Drug Limited by Federal Law to Investigational Use Only** |
| --- |

**9.2 Drug Ordering and Storage**

AT-1501 is manufactured by Lonza and lyophilized and filled by Berkshire Sterile Manufacturing. The filled glass vials will be stored for distribution at Fisher Clinical Services. Initial orders will be addressed to the pharmacy, where the vials for use will be stored.

Instructions on reconstitution, use and storage conditions will be provided in the Pharmacy Manual.

**9.3 Dosage and Administration**

Participants in this study will be enrolled into one of four dose groups: AT-1501 1.0 mg/kg, AT-1501 2.0 mg/kg, AT-1501 4.0 mg/kg, or AT-1501 8.0 mg/kg.

AT-1501 will be given as a 1-hour IV infusion. An IV pump will be used for consistency of delivery. Equipment required for the administration of AT-1501will be described in the Pharmacy Manual and supplied by the clinical site.

A Pharmacy Manual will be provided to the sites giving instructions on reconstitution by dose and by timing of reconstitution as it relates to dose time.

**9.4 Drug Accountability**

The study medication will be kept in a secure area of the pharmacy with limited access. It will be supplied to participants in the trial only under the supervision of the Investigator. The Investigator is responsible for drug accountability and maintaining accurate records of the dispensing of study medication. Drug accountability will be performed by a monitor. Used vials will be kept by the clinical site until drug accountability has been completed by the monitor and returned to Fisher Clinical Services, or if authorized, disposed of at the clinical site and documented.

**10 ADVERSE EVENTS**

**10.1 Definition of Adverse Events**

An AE is any untoward medical occurrence in a patient or clinical investigation participant administered a pharmaceutical product (which does not necessarily have a causal relationship with this treatment) reported during or after having received the investigational product/procedure. An AE can therefore be any unfavorable and unintended sign (including an abnormal laboratory finding), symptom, or disease temporally associated with the use of an investigational product, whether or not related to the investigational product.

**10.1.1 Reporting of Adverse Events**

During the study, the Investigator or clinical site personnel will be responsible for querying and recording adverse events and serious adverse events. Events may be spontaneously reported by the participant, discovered during general questioning by the Investigator, or detected through physical examination, laboratory test or other means. Serious adverse events will be collected from the time of informed consent; all adverse events will be collected from the first administration of investigational product until completion of the final visit and will be evaluated by the Investigator/designee for duration, severity, seriousness, and relationship to investigational product, as outlined below. In addition, the actions taken for the AE will also be documented on the eCRF. Full laboratory data are to be collected in this study, and toxicity trends will be analyzed utilizing objective toxicity criteria.

A baseline recording of any symptoms of illness will be performed before administration of the investigational product. Only symptoms that increase in severity or frequency after investigational product administration or new symptoms of illness will be recorded as AEs. If the event reflects worsening symptoms, then this should be captured in the event term. Untoward medical events which occur after signature of the informed consent but before investigational product administration will be recorded as medical history.

For AEs occurring at the site of infusion the phrase “injection-site” or similar should be included in the event term (e.g. treatment-site erythema). AEs will be evaluated using the Common Terminology Criteria for Adverse Events (CTCAE Version 4.03). All AEs will receive a CTCAE categorization from the Investigator. AEs will also be categorized according to relatedness to the investigational product (see Section 0).

The investigators are responsible for monitoring the safety of patients who have entered this study and for reporting to the Sponsor if serious adverse events (SAE) occur. The Investigator is further responsible for appropriate medical care of patients during the study. The Investigator remains responsible to follow, through an appropriate health care option, AEs that are serious or that caused the patient to discontinue before completing the study. In case of an AE, the patient must be followed until the event resolves or until the Investigator determines the AE has reached a stable outcome or is no longer clinically significant. Frequency of follow-up is left to the discretion of the Investigator. Participant notes throughout the study will be maintained by the Investigator.

**10.1.2 Assessment of Severity of Adverse Event**

All AEs will be assessed using the CTCAE scale as follows:

Grade 1 Mild; asymptomatic or mild symptoms; clinical or diagnostic observations only; intervention not indicated.

Grade 2 Moderate; minimal, local or noninvasive intervention indicated; limiting age-appropriate instrumental Activities of Daily Living (ADL).

Grade 3 Severe or medically significant but not immediately life-threatening; hospitalization or prolongation of hospitalization indicated; disabling; limiting self-care ADL.

Grade 4 Life-threatening consequences; urgent intervention indicated

Grade 5 Death related to AE

**10.1.3 Onset of Adverse Events**

The Investigator will classify the onset of each AE relative to the following stages of treatment:

- During AT-1501 infusion
- After AT-1501 infusion

**10.1.4 Relationship of Adverse Event to Investigational Product**

The Investigator’s assessment of the relationship of the AE(s) to the investigational drug will be assessed as Not Related, Unlikely, Possible or Probable. For these purposes, the following definitions will be used:

**Not Related:** A clinical event, including laboratory test abnormality, with a temporal relationship to drug administration which makes a causal relationship improbable, and in which other drugs, chemicals, or underlying disease provide plausible explanations.

**Unlikely:** A clinical event, including laboratory test abnormality, with a temporal relationship to drug administration which makes a causal relationship unlikely, and in which other drugs, chemicals or underlying disease provide plausible explanations.

**Possible:** A clinical event, including laboratory test abnormality, with a reasonable time sequence to administration of the drug, but which could also be explained by concurrent disease or other drugs or chemicals. Information on drug withdrawal may be lacking or unclear.

**Probable:** A clinical event, including laboratory test abnormality, with a reasonable time sequence to administration of the drug, unlikely to be attributed to concurrent disease or other chemicals, and which follows a clinically reasonable response on withdrawal.

**Related:** An AE that is almost certainly related to the use of the study drug. The AE cannot be reasonably explained by an alternative explanation, e.g., concomitant drug(s), concomitant disease(s).

For purposes of assigning relatedness – Not Related and Unlikely will be considered NOT RELATED. Possible, Probable and Related will be considered RELATED.

**10.2 Serious Adverse Events**

**10.2.1 Definition of a Serious Adverse Event**

A SAE is an adverse event occurring at any dose that results in any of the following outcomes:

Death

Life-threatening

Persistent or significant disability/incapacity

Prompts a hospital admission or prolongs hospitalization

Congenital anomaly/birth defect

Important medical events

Important medical events that may not result in death, be life-threatening, or require hospitalization may be considered serious adverse events when, based upon appropriate medical judgment, they may jeopardize the participant and may require medical or surgical intervention to prevent one of the outcomes listed above. AEs that do not result in any of these outcomes are considered non-serious.

Participants should be closely followed for AEs during the study. Whether or not the adverse events are considered related to the investigational product, they must be reported.

SAEs occurring more than 4 weeks after a patient’s last dose of AT-1501 will NOT be reported unless the Investigator feels that the event may have been caused by the investigational product or a protocol procedure.

Pregnancies occurring within the 3-month period after the last treatment, and coming to the attention of the Investigator, should be followed to document any congenital anomalies or birth defects, should they occur. For purposes of consistency, pregnancies will be recorded in the SAE System; however, the event will then be downgraded if the outcome does not meet the SAE criteria.

**10.2.2 Reporting of Serious Adverse Events**

All SAEs, whether or not thought related to the investigational product, must be reported by the Investigator or designee (by telephone, fax or email) to Safe Harbor Pharmacovigilance within 24 hours of the Investigator becoming aware of the event. The Investigator or responsible clinical site staff will complete and submit the Serious Adverse Event report form and then enter all adverse events into the eCRF.

Serious adverse event reports should be sent to:

Safe Harbor Pharmacovigilance
Phone: +1-984-233-6118
Fax: +1-919-591-0004
Email: safety@safeharborpv.com

Following receipt of the SAE report, the Safety group will review the report and contact the Investigator to request additional information or for data clarification. Redacted medical records supporting the event should be submitted as they become available. For all SAEs occurring during the study, the Investigator must submit follow-up reports to Safe Harbor Pharmacovigilance regarding the participant’s subsequent course until the SAE has resolved, reached a stable outcome, or is no longer clinically significant. The timelines and procedure for follow-up reports are the same as those for the initial report. The submitted reports must be retained by the site.

**10.3 Stopping Rules and Dose Limiting Toxicity (DLT)**

The DLT observation period to determine the safety of a dose is defined as starting with administration of the first dose of AT-1501 to the first participant in a cohort through the administration of the second dose of AT-1501 in the 6th participant in that cohort. This will repeat for each dose level.

Any of the below outlined events occurring during the DLT observation period and considered to be **at least possibly related** to AT-1501 will qualify as a DLT.

A DLT is further defined as:

1. An event which meets the laboratory criteria for potential Drug Induced Liver Injury (DILI) or “Hy’s Law” defined as:

- An elevated alanine transaminase (ALT) or aspartate transaminase (AST) lab value that is greater than or equal to three time (3x) the upper limit of normal (ULN) **WITH**
- An elevated total bilirubin lab value that is greater than or equal to two times (2x) ULN without findings of cholestasis, **AND**
- At the same time, an alkaline phosphatase (ALP) lab value that is less than 2x ULN

1. Any study drug related Grade 3 or greater toxicity with the exception of a Grade 3 or greater laboratory abnormality that is considered clinically insignificant and does not meet the criteria of an adverse event.
2. Any study drug related serious adverse event, regardless of severity.
3. A second occurrence of a similar Grade 2 or greater study drug related event within a cohort. That is, if two or more participants in a cohort experience a similar drug related AE greater than or equal to Grade 2 toxicity, a DLT has been met.

If any DLT criteria are met, further enrollment will be paused and the appropriate DLT data will be reviewed by Anelixis to determine an immediate course of action to be taken. If there are two or more occurrences of a DLT, the study may be temporarily halted until a more thorough safety review is undertaken.

**11 STATISTICAL EVALUATION**

**11.1 Justification of Sample Size**

Approximately 54 adults with ALS will be enrolled into the study. All participants will receive AT-1501. The sample size for this study is not based on formal statistical computation. The number of participants by dose level has been chosen in order to examine initial safety at lower doses and to examine exploratory biomarker endpoints at higher doses with a sufficient number of participants. The sample size may increase in order to allow for replacement of patients who are withdrawn or withdraw prior to receiving at least 4 infusions of AT-1501.

**11.2 Definition of Study Analysis Sets**

**11.2.1 Safety Analysis Set**

All enrolled participants who receive at least one dose of study medication will be included in the Safety Analysis Set (SAS).

**11.2.2 Full Analysis Set**

All enrolled participants who receive at least one dose of study medication and at least one post-baseline measurement will be included in the Full Analysis Set (FAS).

**11.2.3 Evaluable Analysis Set**

The Evaluable Analysis Set (EAS) will include all treated participants who have no important protocol deviations likely to seriously affect the primary outcome of the study.

**11.3 Statistical Analysis**

A detailed description of the planned statistical methods will be documented in a statistical analysis plan (SAP). All statistical analyses will be performed using SAS^®^ software ver. 9.4 or higher.

All continuous variables will be summarized by treatment and visit (as applicable) using descriptive statistics (number of observations [n], mean, median, standard deviation, minimum and maximum). All categorical variables will be summarized by treatment, visit and time point (as applicable) using frequency counts and percentages.

**11.3.1 Study Baseline**

Baseline will be defined as the last available observation prior to administration of the dose.

**11.3.2 Disposition of Participants**

Participant enrollment, inclusion into each study population, discontinuation and withdrawal from the study will be summarized.

**11.3.3 Demography and Baseline Characteristics**

Participant characteristics such as gender, age, race and ethnicity as well as important disease characteristics will be described using summary statistics.

**11.3.4 Safety**

Safety will be summarized by dose level. Adverse events will be mapped by system organ class (SOC) and preferred term using the MedDRA dictionary and will be graded and recorded according to the CTCAE. Safety analyses may include the following:

- Summary of adverse events
- Summary of serious adverse events
- Summary of adverse events by CTCAE grade
- Summary of adverse events related to investigational product
- Summary of adverse events by CTCAE grade and relationship to investigational product
- Laboratory changes from baseline
- Vital signs changes from baseline
- ECG changes from baseline
- C-SSRS changes from baseline
- Percentage of participants to complete all 6 doses

**11.3.5 Clinical Outcome Assessments**

Clinical outcome assessments include ALSFRS-R and spirometry. ALSRFS-R scores will be summed to produce a total score between 0 (worst) and 48 (best). Total scores will be presented with descriptive statistics at each measured timepoint. Changes from baseline will be examined. Spirometry (SVC) measurements will be presented with descriptive statistics at each visit and changes from baseline will be examined, as available.

**11.3.6 Medical History**

Medical history and information about underlying disease for all participants will be listed.

**11.3.7 Prior and Concomitant Medications**

Prior medications are those medications taken before the date of the first dose of investigational product. Concomitant medications are those medications taken on or after the date of the dose of investigational product. All prior and concomitant medications will be summarized by dose level.

**11.4 Handling of Missing Data**

Data will be analyzed as collected. Missing data will not be imputed.

**11.5 Interim Analysis**

No interim analysis is planned for this study.

**11.6 Pharmacokinetic Analyses**

Plasma concentrations of AT-1501 will be listed and a summary by time point for each dose level will be presented; the summary will consist of the number of participants, mean, standard deviation (SD), coefficient of variation (%CV), geometric mean, minimum, median, and maximum. Graphs of the individual subject concentration data and arithmetic mean concentration will be presented.

PK data from the single dose study AT-1501-101 will be used in conjunction with the limited concentration data from the present study to simulate repeat-dose concentration versus time profiles in order to correlate to biomarkers in this study. These analyses will be considered exploratory. Details regarding the analysis and statistical methods will be described in more detail in a separate PK analysis plan for Study AT-1501-A201.

**12 DATA MANAGEMENT**

**12.1 Participant Data Protection**

Participant number, year of birth, race and gender will identify the study subjects in the eCRFs.

The Investigator is responsible for keeping a list of all consented participants including participant numbers, full names and date of birth. In addition, the Investigator will prepare a list of participants who were screened for participation in the trial but were not treated and the reason for non-eligibility.

The participants will be informed in writing that the results will be stored and analyzed in a computer according to national laws, as applicable, and that participant confidentiality will be maintained.

The participants will also be informed in writing about the need for source data verification (SDV), audits and inspections. The audit/inspection and SDV will be performed by at least one of the following parties: authorized representatives of Anelixis Therapeutics, Inc., authorized monitors, clinical site IRBs or regulatory authorities. In these cases, the relevant part of the participant’s notes will be required and reviewed.

**12.2 Data Handling**

The Investigator or his/her designee will document all data obtained during the study using the electronic data capture system provided by Anelixis Therapeutics, Inc. or designee. This also applies to data for those participants who, after having consented to participate, underwent baseline examinations required for inclusion into the trial, but who were not included in the study.

In the process of ensuring data completeness and accuracy, a 100% SDV will be performed. The Investigator will complete and maintain source documents for each participant participating in the study. Source data is the first place the data is recorded, regardless of where this is. AEs and SAEs together with visit dates and information about the disposition for each participant should be recorded in the participant medical chart/notes.

For all participants, Anelixis Therapeutics, Inc. or its designee will validate the completed eCRFs. The Investigator will retain a copy of the printed eCRF, or equivalent. If data management detects eCRFs with missing or inconsistent data, queries will be sent into the system for correction by the Investigator. The Investigator will also receive data queries, which might be generated during the computerized data validation process.

Source data, source documents, CRFs, protocol and amendments, drug accountability forms, correspondence, participant identification list, informed consent forms, and other essential documents must be retained for at least 2 years after the last approval of a marketing application in an ICH region and until there are no pending or contemplated marketing applications in an ICH region, or at least 2 years have elapsed since the formal discontinuation of clinical development of the investigational product. It is the responsibility of the Sponsor to inform the Investigator/institution as to when these documents no longer need to be retained consistent with regulations under ICH GCP.

Anelixis Therapeutics, Inc. or its designee will be responsible for data processing and data control. Participant data will be entered into the clinical database continuously by the clinical site. All original documents and CRFs will be retained in the archives of Anelixis Therapeutics, Inc. as long as the product under investigation remains available for human use.

**13 ADMINISTRATIVE PROCEDURES**

**13.1 Study Completion or Premature Closure**

The Investigator will complete the study and approve the final CRF’s in satisfactory compliance with the protocol within approximately 2 weeks of study completion.

Anelixis reserves the right to close the investigational site or terminate the study at any time. Reasons for the closure of an investigational site or termination of a study by Anelixis may include:

1. Determination of unexpected, significant, or unacceptable risk to patients.
2. Failure to enter patients at an acceptable rate.
3. Insufficient adherence to protocol requirements.
4. Insufficiently complete and/or evaluable data.
5. Plans to modify, suspend or discontinue the development of the study drug.

Should the study be closed prematurely, all study materials (completed, partially completed, and blank CRFs, study drug, etc.) must be returned to Anelixis.

**13.2 Good Clinical Practice**

The study will be conducted in accordance with the current Good Clinical Practice/International Conference on Harmonization (GCP/ICH) Guidelines and relevant regulatory requirement(s). Compliance with this standard provides public assurance that the rights, safety, and well‑being of study participants are being protected consistent with the principles that have their origin in the Declaration of Helsinki and that the clinical study data are credible. The Investigator will be thoroughly familiar with the appropriate use of the investigational product as described in the protocol and Investigator's Brochure. Essential clinical documents will be maintained to demonstrate the validity of the study and the integrity of the data collected. A Trial Master File (TMF) will be established at the beginning of the study, maintained for the duration of the study, and retained according to appropriate regulations.

**13.3 Institutional Review Board**

The trial will be conducted in accordance with the Declaration of Helsinki 1964 including the most recent amendment (Edinburgh, Scotland, October 2000).

The trial protocol, including the participant information and informed consent to be used, must be approved by the appropriate IRB. Written approval must be obtained before enrollment of any participants into the trial. It is the responsibility of the Investigator to supply Sponsor with the Letter of Approval defining the version of each document approved.

The principal Investigator will ensure that this study is conducted in full conformance with the Edinburgh, Scotland, (2000) amendment to the Declaration of Helsinki 1964, 21 CFR part 50, and with national laws and regulations for clinical research.

The Investigator is responsible for informing the IRB and regulatory authorities of any serious adverse events and/or major amendments to the protocol. The Investigator should file all correspondence and a copy should be sent to PRC.

**13.4 Participant Informed Consent**

The Investigator is responsible for giving the participant complete verbal and written information about the nature, purpose, possible risks and benefits of the trial. Trial participants must also be notified that they are free to withdraw from the trial at any time. The participants should have reasonable time to read and digest the information before signing. The Investigator is responsible for obtaining signed IRB approved informed consent from each participant before performing any trial related procedures. In addition to signing the ICF, the participant or legally acceptable representative will be required to authorize access to the participant’s protected health information by signing a separate authorization meeting the requirements of the Health Insurance Portability and Accountability Act of 1996.

A copy of the signed Informed Consent Form will be given to the participant. The signed consent form will be kept by the Investigator in the Investigator Study File.

**13.5 Regulatory Affairs**

This study will be filed under IND 136765. Notifications and reports will be filed according to ICH E6: GCP and FDA guidelines.

**13.6 Trial Monitoring**

Prior to the start of the study, the Monitor will review the protocol, CRFs and other study documents and procedures with the Investigator and their staff. The Investigator will be visited on a regular basis by the Monitor, who will check trial procedures, including safety assessments, drug handling, data recording and SDV. The Monitor will be allowed to review relevant clinical records to confirm that required protocol procedures are being followed and check consistency between participant record and the CRF. Incorrect or missing entries into the CRFs will be addressed as data queries and must be corrected immediately. Trial monitoring will not jeopardize participant confidentiality.

Upon completion of the study the Monitor will make a final assessment of the conduct of the study and inventory all clinical supplies to be returned to Anelixis or designee. Unused or used vials of investigational product will be stored until the Monitor is able to perform a physical inventory and reconciliation with the drug accountability records. At the completion of this study, all unused or used vials must be returned to Anelixis, or designee, or if authorized, disposed of at the study site and documented.

Regulatory authorities, the IRB or IEC and/or Anelixis’ quality assurance group, its CRO designee, or business partners may request access to all source documents, CRFs, and other study documentation for on‑site audit or inspection. Direct access to these documents must be guaranteed by the Investigator, who must provide support at all times for these activities.

**13.7 Trial Audits and Inspections**

During or after the trial has been completed, Anelixis Therapeutics, Inc. representatives or their designee may wish to carry out an audit. Regulatory bodies may also inspect the study. These representatives will have the same access to trial data and participant source data as the Monitor. If a regulatory authority contacts the Investigator with a request for an inspection, the Investigator must inform the Monitor immediately.

**13.8 Financing**

A separate financial agreement (Clinical Trial Agreement) will be signed between Anelixis Therapeutics, Inc. and the Investigator and/or the institution involved.

**13.9 Records Retention**

Essential documents should be retained until at least 2 years after the last approval of a marketing application in an ICH region and until there are no pending or contemplated marketing applications in an ICH region or at least 2 years have elapsed since the formal discontinuation of clinical development of the IP, as described in 21 CFR 312.62. These documents should be retained for a longer period, however, if required by the applicable regulatory requirements or by an agreement with the Sponsor. It is the responsibility of the Sponsor to inform the Investigator/institution as to when these documents no longer need to be retained.

**14 CONFIDENTIALITY AND COMMUNICATION OF RESULTS**

All information concerning Anelixis Therapeutics, Inc. research and product development is considered confidential and will remain the sole property Anelixis Therapeutics, Inc. This includes patent applications, manufacturing processes not previously published and Investigator Brochures.

**14.1 Statistical and Clinical Reports**

Participant listings, including safety data, will be prepared shortly after receiving all participant data. In all listings, participants will be identified by their participant number and initials. Anelixis Therapeutics, Inc. or its designee will also draft and finalize the Clinical Study Report (CSR). If the trial is terminated prematurely for any reason, an abbreviated report will be prepared.

**14.2 Regulatory Use of Data**

By signing the protocol, the investigators agree that the results of this study may be used for submission to national and/or international regulatory agencies. The authorities will be notified of the investigator’s name, address, qualifications and extent of involvement.

**15 REFERENCES**

Boumpas D, Furie R, Manzi S, et al. A short course of BG9588 (Anti-CD40 ligand antibody) improves serologic activity and decreases hematuria in patients with proliferative lupus glomerulonephritis. Arthritis Rheum. 2003 Mar;48(3):719-27

Chamberlain C, Colman PJ, Ranger AM, et al. Repeated administration of dapirolizumab pegol in a randomized phase I study is well tolerated and accompanied by improvements in several composite measures of systemic lupus erythematosus disease activity and changes in whole blood transcriptomic profiles. Ann Rheum Dis. 2017 Nov;76(11):1837-1844. doi: 10.1136/annrheumdis-2017-211388. Epub 2017 Aug 5.

Daikh DI, Finck BK, Linsley PS, et al. Long-term inhibition of murine lupus by brief simultaneous blockade of the B7/CD28 and CD40/gp39 costimulation pathways. J Immunol. 1997 Oct 1;159(7):3104-8.

Durie FH, Aruffo A, Ledbetter J, et al. Antibody to the ligand of CD40, gp39, blocks the occurrence of the acute and chronic forms of graft-vs-host disease. J Clin Invest. 1994 Sep;94(3):1333-8.

Early GS, Zhao W, Burns CM. Anti-CD40 ligand antibody treatment prevents the development of lupus-like nephritis in a subset of New Zealand black x New Zealand white mice. Response correlates with the absence of an anti-antibody response. J Immunol. 1996 Oct 1;157(7):3159-64.

Gerritse K, Laman JD, Noelle RJ et al. CD40-CD40 ligand interactions in experimental allergic encephalomyelitis and multiple sclerosis. Proc Natl Acad Sci U S A. 1996 Mar 19;93(6):2499-504.

Gobburu J, Tenhoor C, Rogge M, et al. Pharmacokinetics/dynamics of 5c8, a monoclonal antibody to CD154 (CD40 ligand) suppression of an immune response in monkeys. J Pharmacol Exp Ther. 1998 Aug;286(2):925-30.

Howard LM, Miga AJ, Vanderlugt CL, et al. Mechanisms of immunotherapeutic intervention by anti-CD40L(CD154) antibody in an animal model of multiple sclerosis. J Clin Invest. 1999 Jan;103(2):281-90.

Kalled SL, Cutler AH, Datta SK, et al. Anti-CD40 ligand antibody treatment of SNF1 mice with established nephritis: preservation of kidney function. J Immunol. 1998 Mar 1;160(5):2158-65.

Kalunian K, Davis J, Merrill J, et al. Treatment of systemic lupus erythematosus by inhibition of T cell costimulation with anti-CD154. Arthritis Rheum. 2002 Dec;46(12):3251-8.

Kanmaz T, Fechner JJ Jr, Torrealba J, et al. Monotherapy with the novel human anti-CD154 monoclonal antibody ABI793 in rhesus monkey renal transplantation model. Transplantation. 2004 Mar 27;77(6):914-20.

Kawai T, Andrews D, Colvin R, et al. Thromboembolic complications after treatment with monoclonal antibody against CD40 ligand. Nat Med. 2000 Feb;6(2):114.

Kim S, Wakwe W, Higginbotham L, et al. Fc-silent anti-CD154 domain antibody effectively prevents nonhuman primate renal allograft rejection. Am J Transplant. 2017 May;17(5):1182-1192.

Koyama I, Kawai T, Andrews D, et al. Thrombophilia associated with anti-CD154 monoclonal antibody treatment and its prophylaxis in nonhuman primates. Transplantation. 2004 Feb 15;77(3):460-2.

Kuwana M, Nomura S, Fujimura K, et al. Effect of a single injection of humanized anti-CD154 monoclonal antibody on the platelet-specific autoimmune response in patients with immune thrombocytopenic purpura. Blood. 2004 Feb 15;103(4):1229-36.

Laman JD, 't Hart BA, Brok H, et al. Protection of marmoset monkeys against EAE by treatment with a murine antibody blocking CD40 (mu5D12). Eur J Immunol. 2002 Aug;32(8):2218-28.

Larsen CP, Alexander DZ, Hollenbaugh D, et al. CD40-gp39 interactions play a critical role during allograft rejection. Suppression of allograft rejection by blockade of the CD40-gp39 pathway. Transplantation. 1996 Jan 15;61(1):4-9.

Lincecum JM, Vieira FG, Wang MZ, et al. From transcriptome analysis to therapeutic anti-CD40L treatment in the SOD1 model of amyotrophic lateral sclerosis. Nat Genet. 2010 May;42(5):392-9. doi: 10.1038/ng.557. Epub 2010 Mar 28.

Mohan C., Shi Y., Laman J.D., et al. Interaction between CD40 and its ligand gp39 in the development of murine lupus nephritis. J Immunol. 1995 Feb 1;154(3):1470-80.

Parker DC, Greiner DL, Phillips NE, et al. Survival of mouse pancreatic islet allografts in recipients treated with allogeneic small lymphocytes and antibody to CD40 ligand. Proc Natl Acad Sci U S A. 1995 Oct 10;92(21):9560-4.

Samoilova EB, Horton JL, Zhang H, et al. CD40L blockade prevents autoimmune encephalomyelitis and hampers TH1 but not TH2 pathway of T cell differentiation. J Mol Med (Berl). 1997 Aug;75(8):603-8.

Schaub M, Issazadeh S, Stadlbauer TH, et al. Costimulatory signal blockade in murine relapsing experimental autoimmune encephalomyelitis. J Neuroimmunol. 1999 May 3;96(2):158-66.

Schuler W, Bigaud M, Brinkmann V, et al. Efficacy and safety of ABI793, a novel human anti-human CD154 monoclonal antibody, in cynomolgus monkey renal allotransplantation. Transplantation. 2004 Mar 15;77(5):717-26.

Shock A, Burkly L, Wakefield I, et al. CDP7657, an anti-CD40L antibody lacking an Fc domain, inhibits CD40L-dependent immune responses without thrombotic complications: an in vivo study. Arthritis Res Ther. 2015 Sep 3;17:234.

Tan J, Town T, Crawford F, et al. Role of CD40 ligand in amyloidosis in transgenic Alzheimer's mice. Nat Neurosci. 2002 Dec;5(12):1288-93.

Town T, Tan J, Mullan M. CD40 signaling and Alzheimer's disease pathogenesis. Neurochem Int. 2001 Nov-Dec;39(5-6):371-80.

Wakefield I, Harari O, Hutto D, et al. An assessment of the thromboembolic potential of CDP7657, a monovalent Fab' PEG anti-CD40L antibody, in rhesus macaques. Arthritis Rheum. 2010 Jan;62:1243.

Wang X, Huang W, Mihara M, et al. Mechanism of action of combined short-term CTLA4Ig and anti-CD40 ligand in murine systemic lupus erythematosus. J Immunol. 2002 Feb 15;168(4):2046-53.

**Appendix A** **Schedule of Events**

|  | **Screening** | **Dosing** | | | **Telephone Check** | **Dosing** | **Follow-up** |
| --- | --- | --- | --- | --- | --- | --- | --- |
| **Visit** | **1** | **2** | | | **3, 5, 7, 9, 11, 13** | **4, 6, 8, 10, 12** | **14** |
| **Study Week** | **Weeks -4 to -1** | **Week 1** | | | **Weeks 2, 4, 6, 8, 10 and 12** | **Weeks 3, 5, 7, 9 and 11** | **Week 15** |
| **Study Day** | **Day -30 to -1** | **Day 1 Pre-dose** | **Day 1 Dose** | **Day 1 Post-dose** | **Days 8, 22, 36, 50, 64, 78**  **(+/-2 days)** | **Days 15, 29, 43, 57, 71**  **(+/-2 days)** | **Day 101**  **(+/-7days)** |
| Informed Consent | X |  |  |  |  |  |  |
| Medical History and Demographics | X |  |  |  |  |  |  |
| Record Concomitant Medications | X | X | X | X | X | X | X |
| Review Eligibility Criteria | X | X |  |  |  |  |  |
| Physical Examination^1^ | X | X |  |  |  | X | X |
| Vital Signs^2^ | X | X | X | X |  | X | X |
| Weight^3^ | X |  |  |  |  | X |  |
| Height | X |  |  |  |  |  |  |
| ECG^4^ | X |  |  | X^4^ |  | X^4^ | X |
| COVID-19 testing^14^ | X |  |  |  |  |  |  |
| Blood/Urine Samples for Eligibility/Safety Testing ^5, 6, 7^ | X^5^ | X^6,7^ |  |  |  | X^6,7^ | X^6^ |
| Blood Samples for Immunogenicity^8^ |  | X^8^ |  |  |  | Weeks 3 and 5^8^ (pre-dose) | X |
| Blood Samples for AT-1501 Concentration^9^ |  | X^9^ |  | X^9^ |  | X^9^  (pre and post dose) | X |
| Blood Samples for Biomarker Analysis^10^ | X | X^10^ |  |  |  | X^10^  (pre-dose) | X |
| ALSFRS-R^11^ | X | X |  |  |  | X | X |
| Spirometry^12^ | X | X |  |  |  | Weeks 5, 9, 11 (pre-dose) | X |
| Colombia Suicide Severity Rating Scale | X | X |  |  |  | X (pre-dose) | X |
| AT-1501 1-hr Infusion^13^ |  |  | X |  |  | X |  |
| Report Serious Adverse Events only | X | X |  |  |  |  |  |
| Assess for All Adverse Events |  |  | X | X | X | X | X |

1. Complete physical examination at Screening; symptom-directed physical examination at other times.
2. Vital signs (blood pressure, heart rate, temperature, respiration rate) are taken after being seated/supine for 5 minutes at Screening and then pre-dose, 15 minutes into the infusion and 1 and 2 hours post-infusion. Assessments performed post-infusion are timed from the end of the 1-hour infusion, not the IV-line flush. Vital signs may be collected within +5 min of the 15 minute timepoint and +15 min of the 1 and 2 hour post-infusion timepoints.
3. Weight for dose calculation is taken at Screening and on each dosing day pre-infusion. A weight change of 10% or greater will warrant a recalculation of the AT-1501 dose.
4. Post-infusion ECGs should be read on the day they are taken before the participant is released.
5. Eligibility testing includes serum chemistry, CBC with differential and platelet count, coagulation tests, serum pregnancy, HIV, HBC, HCV and urinalysis
6. Safety testing includes serum chemistry, CBC with differential and platelet count, coagulation tests, urinalysis and urine pregnancy test
7. Blood samples for safety testing pre-dose on dosing days only.
8. Blood samples for immunogenicity testing pre-dose only on weeks 1, 3 & 5 and end of study visit.
9. Plasma for AT-1501 concentration are pre-dose and 2 hours after the start of the infusion dose.
10. Blood samples for biomarker analysis are done at screening and pre-dose on all dosing days.
11. ALSFRS-R is performed at screening and pre-dose on each dosing visit and at the end of study visit.
12. Spirometry will be measured at Screening and pre-dose at weeks 1, 5, 9 &11, where and when it is available.
13. AT-1501 to be infused over 1 hour using an IV pump. Assessments performed post-infusion are timed from the end of the 1-hour infusion, not the IV-line flush.
14. A COVID-19 test will be conducted at screening via nasal swab no longer than 1 week prior to their infusion. If a patient arrives at the treatment center and is symptomatic for COVID-19 subsequent testing may be administered to determine continued study eligibility.
